# AI for prognostic assessment of diffuse large B‐cell lymphoma using H&E whole‐slide images

**DOI:** 10.1002/hem3.70463

**Published:** 2026-08-31

**Authors:** Jonas Lippl, Sarah Reinke, Stefan Schrod, Lukas Wolfseher, Paul Hüttl, Michael Huttner, Annette M. Staiger, Ilske Oschlies, Karoline Koch, Edward G. Michaelis, Julia Richter, Hilka Rauert‐Wunderlich, Nicole Seifert, Robin Kosch, Norbert Schmitz, Marita Ziepert, Lorenz Thurner, Viola Poeschel, Bertram Glaß, Lorenz Trümper, Gerhard Held, David W. Scott, Andreas Rosenwald, German Ott, Rainer Spang, Michael Altenbuchinger, Wolfram Klapper

**Affiliations:** ^1^ Department of Medical Bioinformatics University Medical Center Göttingen Göttingen Germany; ^2^ Department of Pathology, Hematopathology Section University Hospital Schleswig‐Holstein, Campus Kiel Kiel Germany; ^3^ Department of Statistical Genomics and Systems Genetics European Molecular Biology Laboratory (EMBL) Heidelberg Germany; ^4^ Department of Statistical Bioinformatics University of Regensburg Regensburg Germany; ^5^ Department of Pathology Robert Bosch Hospital Stuttgart Stuttgart Germany; ^6^ Dr Margarete Fischer‐Bosch Institute of Clinical Pharmacology Stuttgart Germany; ^7^ University of Tuebingen Tübingen Germany; ^8^ Department of Pathology Charité University Hospital Berlin Berlin Germany; ^9^ Institute of Pathology University of Würzburg Würzburg Germany; ^10^ Peter L. Reichertz Institute for Medical Informatics TU Braunschweig Braunschweig Germany; ^11^ Hannover Medical School Hannover Germany; ^12^ Medical Clinic A, University Hospital Münster Münster Germany; ^13^ Department of Medical Informatics, Statistics and Epidemiology University of Leipzig Leipzig Germany; ^14^ Department of Internal Medicine I University Hospital of Saarland Homburg Germany; ^15^ Department of Hematology, Oncology, and Tumor Immunology Helios Klinikum Berlin‐Buch Berlin Germany; ^16^ Department of Hematology, Oncology Georg August University of Göttingen Göttingen Germany; ^17^ Department of Internal Medicine Westpfalz‐Klinikum Kaiserslautern Germany; ^18^ BC Cancer and University of British Columbia Vancouver British Columbia Canada

## Abstract

Prediction of the outcome of large B‐cell lymphomas/high‐grade B‐cell lymphomas (DLBCLs/HGBCLs) is based on clinical parameters and molecular testing, for example, for rearrangements of MYC (MYC‐R) and MYC‐R in combination with BCL2 and BCL6 translocations (double/triple hit). However, the group of DLBCL/HGBCL with poor outcome is not confined to MYC‐R lymphomas, and fluorescence in situ hybridization (FISH) testing for MYC‐R misses several high‐risk lymphomas. We aimed to understand if artificial intelligence (AI) trained to identify MYC‐R will delineate a poor prognostic subgroup. We generated a collection of digital hematoxylin and eosin (H&E)‐stained slides of DLBCL/HGBCL (*N* = 2018) annotated for MYC, BCL2, and BCL6 translocations. A multiple‐instance deep learning AI model for the identification of MYC‐R alone or as double/triple hit was established using 1035 H&E‐stained slides and evaluated on an external test cohort (*N* = 499). A pretrained tumor tissue classifier improved reliability and interpretability by focusing the model on tumor areas. Our model score reflects a morphological “MYCness” in DLBCL/HGBCL and demonstrates a strong association with overall survival (OS) and progression‐free survival (PFS) in the external test cohort. This was confirmed with an additional clinical test cohort (*N* = 484) without FISH labels. The AI model scores correlate with various molecular features of DLBCL/HGBCL, including BCL2 and MYC gene expression, and the high‐grade gene expression signature, but also features of the tumor microenvironment. Multivariate analysis, adjusted for International Prognostic Index (IPI) factors, demonstrated the prognostic significance of our model in identifying high‐risk cases for both PFS and OS.

## Graphical abstract

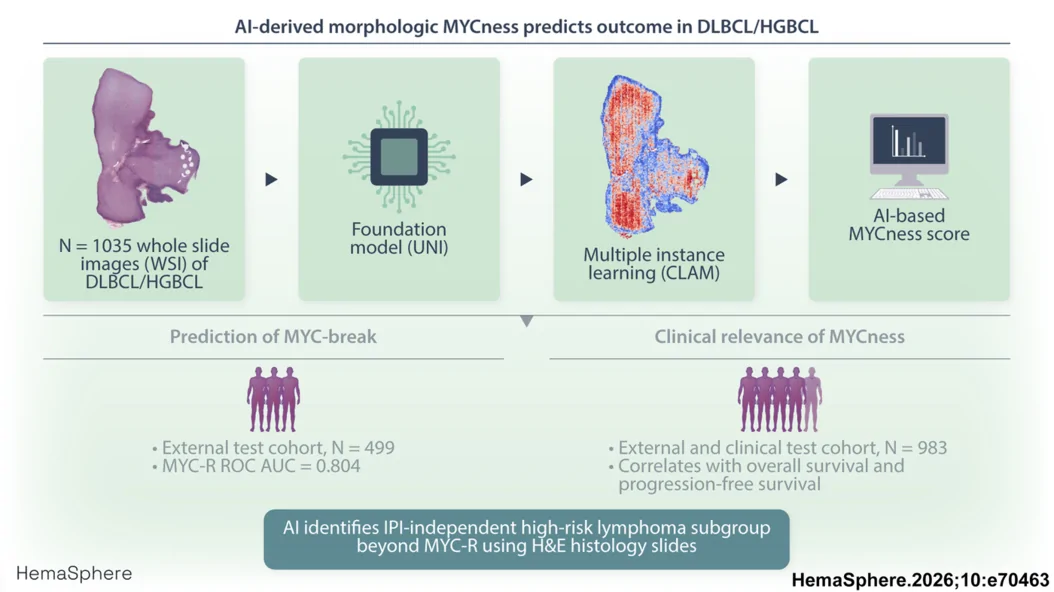

## INTRODUCTION

A subgroup of large B‐cell lymphomas (DLBCLs) harbors translocations involving the *MYC* gene (MYC‐rearranged, MYC‐R). These lymphomas are either classified as diffuse DLBCL or high‐grade B‐cell lymphoma (HGBCL) depending on the morphology and additional genetic features such as the co‐occurrence of *BCL2* and *BCL6* translocations as double hit (DH) and triple hit (TH), respectively.^1^, ^2^ The formal classification and nomenclature of DH and TH lymphomas differ slightly between the WHO^1^ and the ICC^2^ classifications. In this study, all DLBCLs are collectively referred to as DLBCL/HGBCL. Approximately 10% of lymphomas with the DLBCL/HGBCL morphology carry MYC breaks/translocation and are characterized by an unfavorable outcome.^3^ Consequently, screening of DLBCLs for MYC translocations by fluorescence in situ hybridization (FISH) has become a standard diagnostic procedure.^1^, ^2^, ^4^ Once a DLBCL/HGBCL turns out to be MYC‐R, the specimen is usually subsequently tested for BCL2 and BCL6 translocations.^5^

However, single‐gene molecular assays such as FISH to identify prognostically unfavorable DLBLC/HGBCL do not appear to have a promising future in DLBCL/HGBCL risk stratification for several reasons. First, molecular testing is not ubiquitously available in pathology laboratories, which may lead to delays in diagnosis and may also result in considerable costs. Thus, attempts have been undertaken to screen for cases by immunohistochemistry for MYC protein and restricting subsequent FISH analysis to a limited number of biopsies.^6^, ^7^ Despite these efforts, it is now generally accepted that the presence of MYC translocations cannot be reliably detected by MYC immunohistochemistry under common diagnostic conditions.^8^ Second, and most importantly, the group of MYC‐R/DH/TH hit lymphomas comprises only a subgroup of DLBCL/HGBCL with poor outcome, and FISH testing misses a relevant number of cases that fail under standard therapy.^3^ However, DH/TH DLBCL/HGBCL have successfully been used as a blueprint to identify lymphomas with a high risk of relapse using gene expression profiling for a “double‐hit signature,” which also identifies lymphomas without MYC‐R with high risk.^9^, ^10^ Unfortunately, this technology is not widely available in most diagnostic labs.

A cost‐efficient method is required to detect MYC‐R as a surrogate for high‐risk lymphomas in laboratories without expertise in molecular biology or access to the necessary technology, given the clinical need to identify the poor prognostic subgroup. One obvious approach to meeting this medical need is AI‐based MYC‐R detection from hematoxylin and eosin (H&E)‐stained slides, which are generated for all patients by default during the diagnostic procedure. Deep learning methods are a key component of AI. The application of deep learning to whole‐slide images (WSIs)—known as computational pathology—is a rapidly evolving field. Applications of computational pathology to H&E WSIs include tumor detection,^11^, ^12^ survival analysis,^13^, ^14^, ^15^, ^16^, ^17^ or tumor subtyping.^18^ Recent approaches have overcome limitations such as a lack of annotations at the pixel (px) level^19^ or access to graphics processing units (GPUs).^20^ Multiple‐instance learning (MIL), a weakly supervised approach, utilizes the WSI label (diagnosis) to determine patches of the slide that are most relevant for the diagnosis instead. This approach has been the most successful for pathology image analysis.^20^, ^21^, ^22^ However, despite this progress in digital pathology, success in detecting a single genetic aberration such as MYC‐R in DLBCL/HGBCL analyzing H&E WSI is limited.^23^, ^24^, ^25^, ^26^, ^27^ Shortcomings of published studies include a high false‐positive detection rate (AI predicting MYC in cases that do not harbor MYC‐R) and a lack of clinical testing of these models.^23^, ^24^, ^25^, ^26^, ^27^

In the current study, we investigated whether the false‐positive rate of AI detecting MYC on H&E WSI can be reduced using the so far largest cohort of DLBCL/HGBCL H&E WSI and a novel filtering step, ensuring that AI models focus on lymphoma areas. However, our main goal was to understand whether the AI‐based MYC‐R prediction as a continuous score of morphological “MYCness” could identify clinically aggressive disease, similar to the high‐grade signature by gene expression profiling. Thus, we tested the AI score as a continuous parameter for its prognostic power in patients treated in prospective clinical trials.

## MATERIALS AND METHODS

This study was designed in accordance with the standards for reporting clinical prediction models (Supporting Information S2: TRIPOD + AI Statement).

### Whole‐slide images and genetic annotations

**Figure 1:**
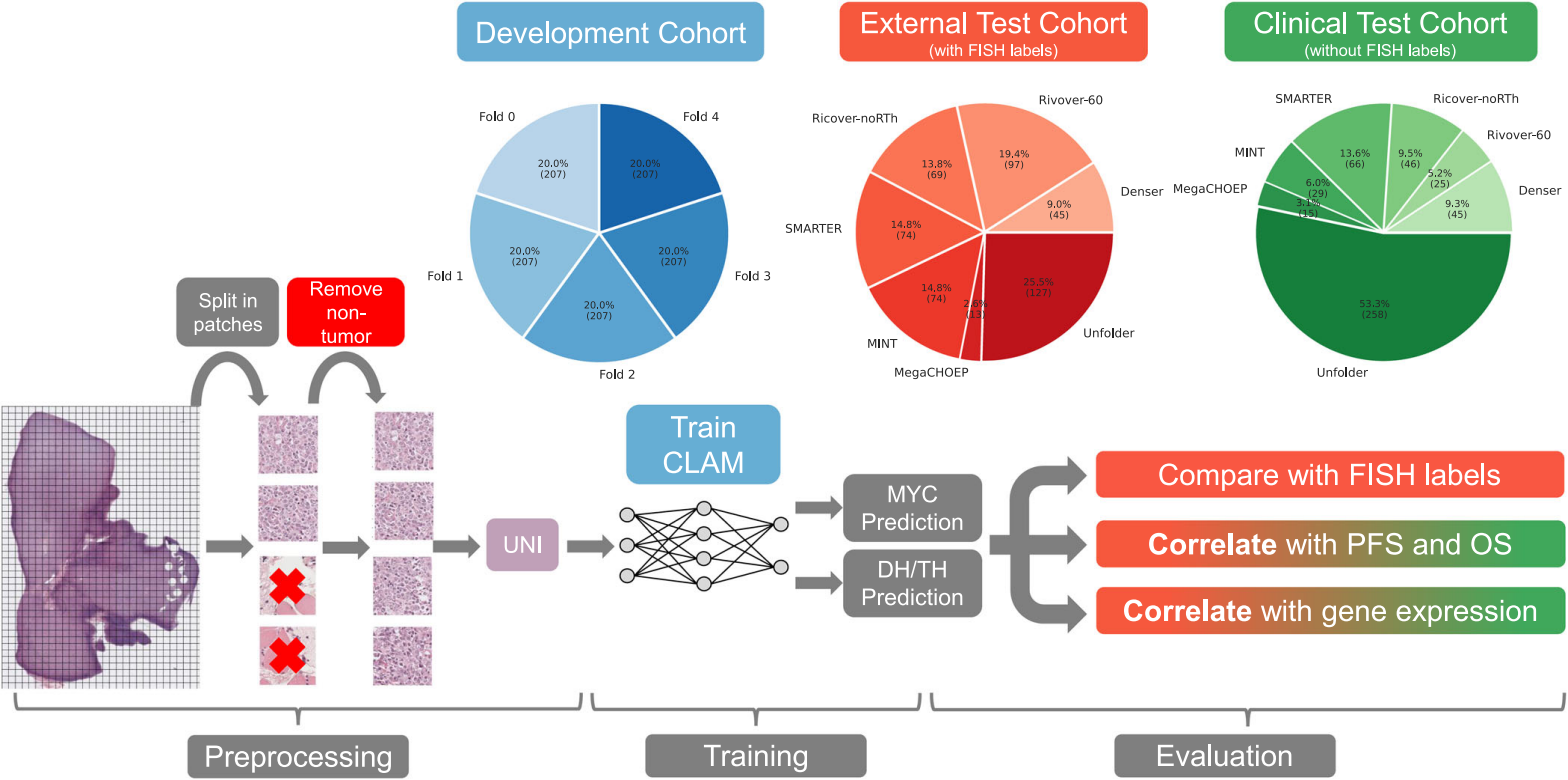
**Workflow of this paper.** Six multiple‐instance learning (MIL) architectures are benchmarked, with CLAM providing the best performance. Predicted scores are subsequently evaluated on fluorescence in situ hybridization (FISH) labels of the external test cohort and correlated with clinical data of the external and clinical test cohort. Furthermore, correlations between gene expression values and the double‐hit signature were calculated for a subgroup of patients of the external test cohort and the clinical test cohort with available data. DH, double hit; OS, overall survival; PFS, progression‐free survival; TH, triple hit.

The overall workflow of our study is illustrated in Figure 1. Three cohorts of H&E WSI pathologically reported as DLBCL or HGBCL specimens were generated to enable the development of a deep learning‐based histomorphological score for the presence of MYC‐R and/or DH/TH and to evaluate its association with clinical outcome (Supporting Information S1: Table 1). The first cohort, referred to as the development cohort, comprises *N* = 1035 DLBCL/HGBCL specimens, collected between 2000 and 2023 from the files of the Hematopathology Section in Kiel, Germany. Each case was reviewed by expert hematopathologists and tested for MYC‐R using FISH by the break‐apart assay, as previously described.^28^ Lymphomas showing MYC‐R were further analyzed for breaks in BCL2 and BCL6 to differentiate between MYC‐R, DH, and TH lymphomas, and all categories served as molecular reference labels for model training.^3^ For the development cohort, no clinical follow‐up was available. The second and third cohorts were established from patients enrolled in several prospective clinical trials of the German High Grade Lymphoma Study Group/German Lymphoma Alliance (GLA), including Denser^29^ (*N* = 90), MINT^30^ (*N* = 103), MegaCHOEP^31^ (*N* = 28), Ricover‐60^32^ (*N* = 122), Ricover‐noRTh^33^ (*N* = 115), Smarter^34^ (*N* = 141), and Unfolder^35^ (*N* = 385). These two test cohorts consisted of a total of *N* = 983 patients treated with immunochemotherapy, including anti‐CD20 antibodies. Follow‐up information was available for these patients regarding overall survival (OS) and progression‐free survival (PFS), as well as clinical data such as the International Prognostic Index (IPI), enabling an independent evaluation of the prognostic relevance of the model score. Within these two test cohorts, FISH annotations were available for *N* = 499 lymphomas^36^, ^37^ (external test cohort). The remaining 484 cases with available WSIs and clinical data, but lacking genetic annotations, were referred to as the “Clinical Test Cohort” (Figure 1). The number of WSIs was slightly higher than the number of patients mentioned above, because approximately 100 patients in the external test cohort had multiple images available. In these cases, predictions were averaged across images, and only one value per patient was used for further analysis. For study protocols, register numbers, and other details of the clinical trials setting, refer to their original publications.^29^, ^30^, ^31^, ^32^, ^33^, ^34^, ^35^ The development cohort was enriched for MYC‐R and DH/TH cases, whereas the external and clinical test cohorts represent the prevalence of these genetic alterations in adults in Central Europe.^38^

### Data preprocessing

WSIs were converted from TIFF files into an SQLite database Pamly,^39^ an open‐source, in‐house‐developed library built on OpenSlide.^40^ Only tiles containing tissue were stored, and white background areas were removed. Tiles measuring 512 × 512 pixels were sampled at the highest resolution, which corresponds to a magnification level of 400× of a conventional light microscope. For each tile, a 1024‐dimensional feature embedding was generated using the pretrained model known as UNI.^41^ UNI is a Vision Transformer^42^ that was pretrained in a self‐supervised manner on 100,000 WSIs from various tissue types. The full data preprocessing pipeline can be found at https://gitlab.gwdg.de/jlipl/cpathpipeline. To facilitate tumor tissue classification, we curated a set of *N* = 28,000 patches that were manually annotated by expert pathologists. The tumor patch labels included “Tumor,” “Tumor, Poor Quality,” and “Partial Tumor” for tumor‐like patches, while non‐tumor patches were labeled as “Fatty Tissue,” “Connective Tissue,” and others (Supporting Information S1: Supplementary Methods). These labeled patches were used to train a tumor classifier, which was subsequently used to generate tumor probability maps and to pre‐filter patches for downstream classification tasks.

### Model development and validation

In total, we evaluated six different MIL methods on the training data using a fivefold cross‐validation, to identify a model capable of learning robust histomorphological representations associated with aggressive lymphoma biology. The MYC‐R and DH/TH status served as training labels. Tested methods were ABMIL,^21^ CLAM,^20^ TransMIL,^22^ AttriMIL,^43^ Snuffy,^44^ and DTFD‐MIL^45^ (Supporting Information S1: Supplementary Methods). The best model was selected based on the area under the curve (AUC) of the receiver operating characteristic (ROC) in cross‐validation using the training data and subsequently tested on the external test cohorts (*N* = 499). Due to the imbalance in the classes (Supporting Information S1: Table 1), balanced accuracy was used as a robust, unbiased metric to establish the thresholds for the dichotomized classification score. To determine if direct optimization for survival could further enhance prognostic performance, the best performing model was additionally fine‐tuned on the external test cohort using DeepSurv^46^ loss for both OS and PFS. The resulting fine‐tuned model was subsequently evaluated on the independent clinical test cohort.

### Clinical and molecular analyses

We assessed the correlation between the score derived from the selected model and OS or PFS by stratifying patients into two or four groups using the optimal threshold or quartiles, respectively. Kaplan–Meier (KM) curves were then used to compare OS and PFS probabilities among the subgroups. A log‐rank test was used to calculate P‐values and the concordance index (*C*‐index) was used to quantify the correlation. The relationship between risk factors, trial, treatment, cell‐of‐origin (COO) subtype, and outcomes was determined using a Cox proportional hazards (CoxPH) model. Likelihood ratio tests (LRTs) were used to evaluate the independent prognostic contribution of the model score. The correlation between molecular features such as gene expression and the model score was evaluated using Pearson's correlation coefficient.

## RESULTS

### Model development and primary validation

**Figure 2:**
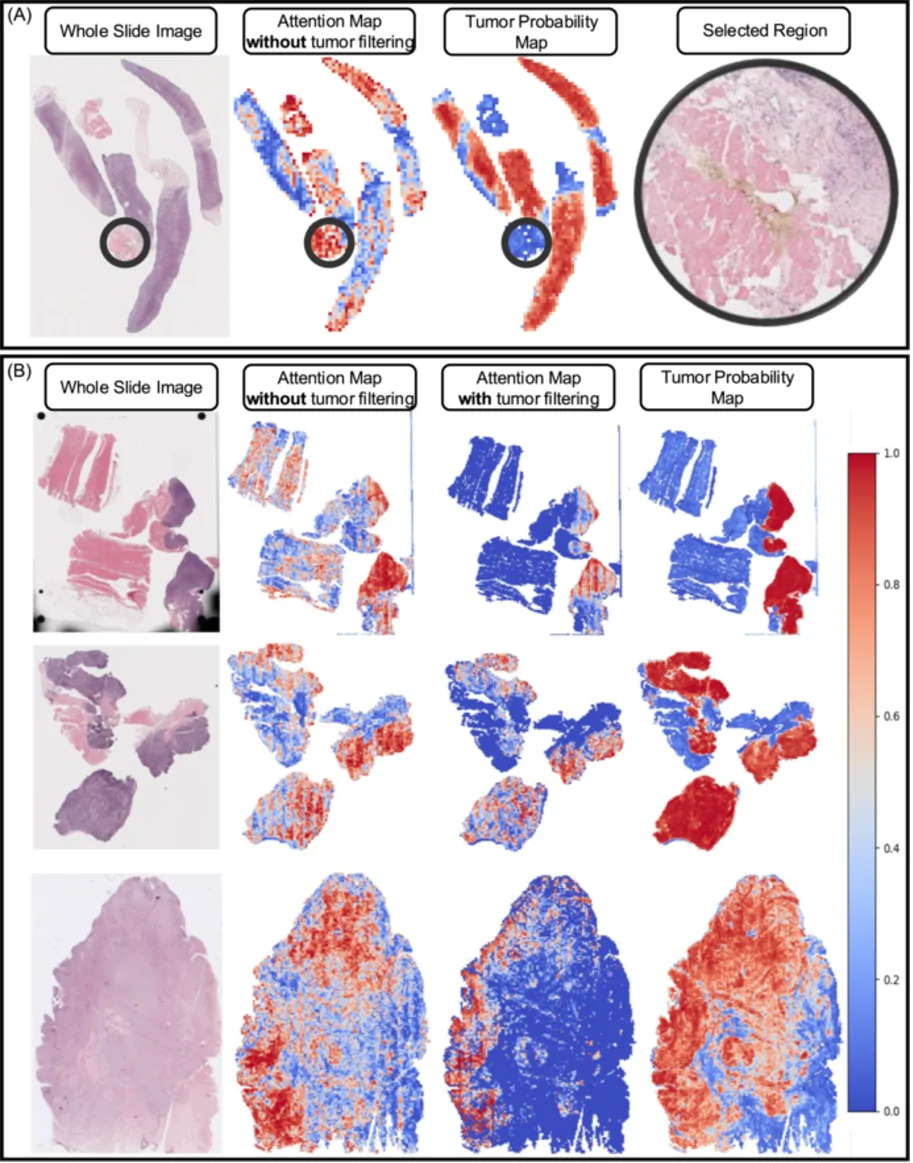
**Illustration of attention scores across different image regions. (A)** Detailed illustration of non‐tumor regions with unwanted high attention scores (visualized as percentiles per slide) by the model. The selected region in the hematoxylin and eosin (H&E)‐stained whole‐slide image (WSI) (black circle) receives high attention scores by the model, but has a very low tumor probability. High magnification reveals skeletal muscle and connective tissue, but no lymphoma. **(B)** The figure consists of three WSIs, each shown in a separate row: the original H&E‐stained WSI, the model's attention map without tumor pre‐filtering, the model's attention map after tumor pre‐filtering, and the tumor probability map. The color scale on the right indicates the respective score, ranging from red (high) to blue (low). Without pre‐filtering, high attention is assigned to eosinophilic areas, which represent smooth muscle tissue (upper row) or necrosis (middle row).

In total, six deep learning architectures were developed to learn histomorphological patterns associated with aggressive disease biology in DLBCL, using MYC‐R and DH/TH status as training labels. A comprehensive benchmark analysis was performed for model selection using fivefold cross‐validation on the development cohort of *N* = 1035 DLBCL/HGBCL (Figure 1 and Supporting Information S1: Table 1). CLAM^20^ yielded the best performance, with a cross‐validation ROC AUC (Supporting Information S1: Figure 1) of 0.824 ± 0.036 for MYC‐R classification, and was selected as the final model (Supporting Information S1: Table 2). The corresponding performance evaluation in terms of area under the precision recall curve (AUPRC) supports this choice (Supporting Information S1: Table 2). For consistency, models for DH/TH prediction were established using the same workflow including a benchmark analysis (Supporting Information S1: Table 3). The latter confirmed CLAM to be one of the most suitable training strategies. Decision thresholds of 0.30 and 0.19 were finally established according to the maximal balanced accuracy of 0.75 (Supporting Information S1: Figure 2A) and 0.77 for MYC‐R and DH/TH classification, respectively. A model that only utilized MYC/BCL2‐DH lymphomas showed a similar performance (Supporting Information S1: Table 4).

Visualization of the models was performed by extraction of attention maps according to the procedure of Lu et al^20^ (Figure 2). This visualization indicated that the model assigns high attention to areas of the WSI, which do not represent well‐preserved lymphoma tissue according to a pathologist's visual evaluation (Figure 2A). To improve the plausibility of AI predictions, we restricted attention maps to regions identified by an expert pathologist‐trained lymphoma pre‐filtering algorithm. The resulting new visualization successfully focuses attention on regions likely containing tumor cells, while image artifacts, such as blurred patches and those at the boundary of the tissue specimen, are excluded (Figure 2B). It is worth noting that restricting the analysis to tiles containing lymphoma tissue and excluding irrelevant areas according to our pathologist‐pretrained algorithm did not improve the model's ability to predict MYC‐R when applied prior to the training (Supporting Information S1: Table 2). Nevertheless, the algorithm generates a result that enables pathologists to understand how the model works and likely increases confidence in the results.

### External testing

**Figure 3:**
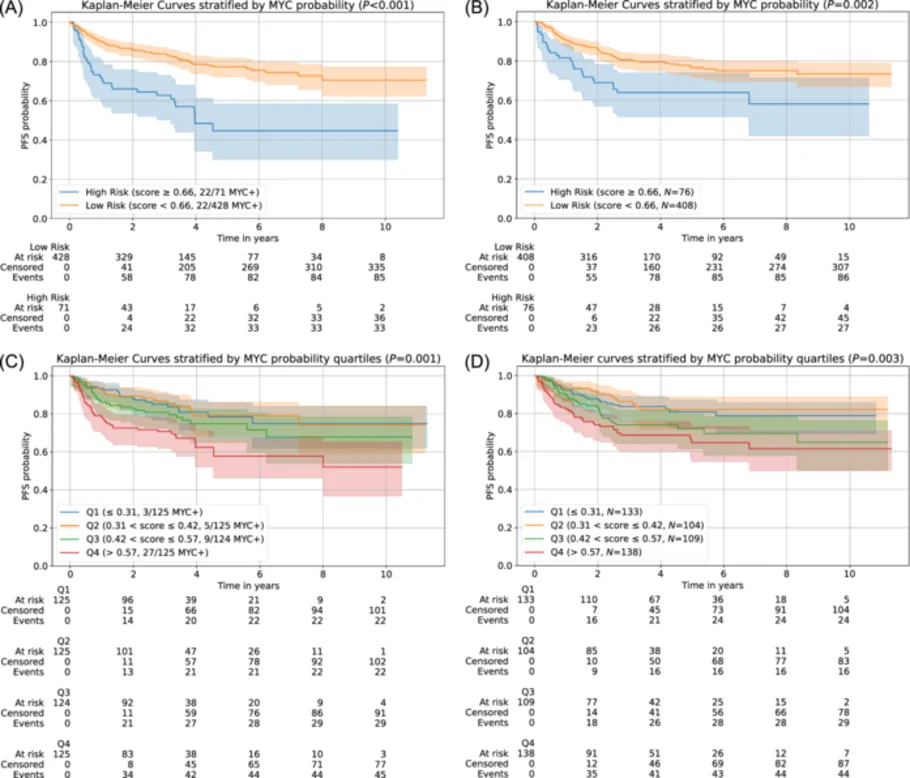
**Kaplan–Meier curves for progression‐free survival.** Kaplan–Meier curves for progression‐free survival (PFS) of the external test cohort **(A, C)** and the clinical test cohort **(B, D)** for patient stratification based on model scores. The numbers of MYC‐R cases and total patients in each subgroup are listed in the legends. First, the threshold for stratification was selected using the minimum P‐value on the external test cohort **(A)** and applied to the clinical test cohort **(B)**. In addition, patients are stratified using quartiles of the model scores **(C**, **D)**.

The established models for MYC‐R and DH/TH were subsequently tested using the external test cohort that included WSIs from *N* = 499 cases with corresponding genetic annotations (workflow Figure 1). Our AI models for MYC‐R and DH/TH prediction were confirmed with an ROC AUC of 0.804 (Supporting Information S1: Figure 1 and Supporting Information S1: Table 2) and 0.778, respectively (Supporting Information S1: Table 3). The established decision thresholds yielded balanced accuracies of 0.75 (Supporting Information S1: Figure 2A) and 0.54, respectively. With these thresholds, our model predicted 41/44 (93%) MYC‐R (true positive) and missed 3/44 (7%) cases with MYC‐R by FISH as false negative. Notably, 319/455 (70%) of cases without MYC‐R by FISH showed a high model score above the applied threshold, resulting in poor model calibration (Supporting Information S1: Figure 3). This finding suggests that the model score to which we refer as morphological “MYCness” is high in a subgroup of DLBCL/HGBCL without MYC‐R/DH/TH.

### Clinical relevance

To ensure that the model focused on tumor morphology, we used the tumor‐filtered model for all subsequent analyses. Since our model score is the highest in lymphomas with MYC‐R but also frequently increased in lymphomas lacking MYC‐R, we reasoned that despite training for MYC‐R, our model score reflects AI‐detectable morphological features (“MYCness”) shared by a group of lymphomas with and without MYC‐R. In analogy to the gene expression high‐grade signature, we hypothesized that a high score of morphological “MYCness” should be associated with poor outcome independent of the presence of MYC‐R. Thus, we tested for an association of model scores with PFS and OS on the external test cohort via Cox regression, in which the respective model score serves as a continuous predictor variable (Supporting Information S1: Table 5). The most significant association was found between the MYC‐R prediction score and PFS and OS (P < 0.001) even exceeding the prognostic value of the genetic status as MYC‐R/DH/TH assessed by FISH. It is noteworthy that the MYC‐R prediction score showed a stronger association with PFS and OS compared to the DH/TH score (Supporting Information S1: Table 7). To further optimize the model for predicting OS and PFS, we fine‐tuned it by training the final layers with DeepSurv^46^ loss on the external test cohort. However, the fine‐tuned model to the independent clinical test cohort did not provide any additional prognostic information beyond the original MYC‐R score. The *C*‐index for OS and PFS was the same or slightly lower for the original and fine‐tuned models. Therefore, we conclude that training an AI‐based MYC‐R score already achieved a prognostic performance that could not be further improved by fitting with the clinical outcome in the available data set. Therefore, the MYC‐R score was used for all subsequent analyses.

**Figure 4:**
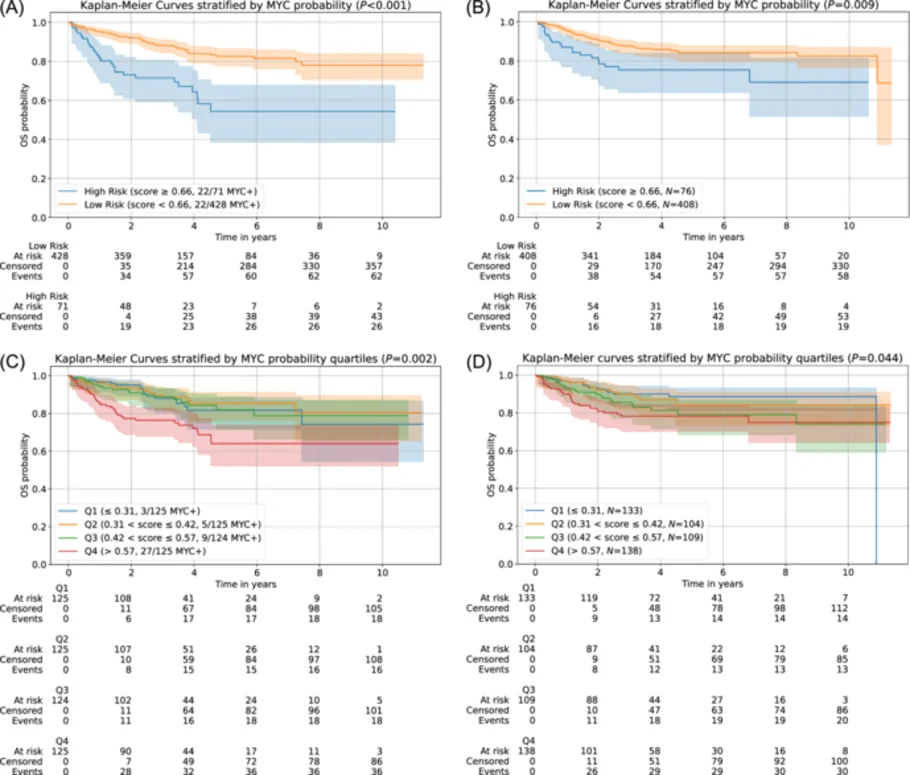
**Kaplan–Meier curves for overall survival.** Kaplan–Meier curves for overall survival (OS) of the external test cohort **(A, C)** and the clinical test cohort **(B, D)**. Risk groups were defined based on model scores, using a cutoff selected via minimum P‐value analysis of the external test cohort **(A)**, and then applied to the clinical test cohort **(B)**. Quartiles **(C**, **D)** were used for additional visualization and patient stratification. The numbers of MYC‐R cases and patients in each subgroup are provided in the legends.

To explore potential confounding variables, additional Cox regression analyses including international prognostic score (IPI) risk groups (low, low‐intermediate, high‐intermediate, and high), the trial, treatment, and COO subtype as additional predictor variables were conducted. In the analysis including only IPI as an additional variable, the model score retained significance (P‐values 0.002 for OS and <0.001 for PFS). Interestingly, the prognostic relevance of the model score for PFS also remained significant when the respective ground‐truth genetic annotation (the FISH result) was included in the Cox analysis as an independent variable (Supporting Information S1: Table 5, Supporting Information S1: Figure 4). Significance was also retained for the remaining analyses, including those for trial, treatment, and COO subtypes. This finding indicates that the trained score identifies histological features that are more closely linked to clinical aggressiveness than the objective of learning, MYC‐R, itself. This is also confirmed by a stratification within the MYC− and MYC+ subgroups, in which the model can still identify significant low‐ and high‐risk patients (Supporting Information S1: Figure 5). Furthermore, in another Cox regression analysis, we confirmed that the score is not confounded by the mere identification of trials or treatment groups. To verify the results on independent data, the model was applied to the clinical test cohort, which included patients from prospective clinical trials for which genetic annotations were not available. The Cox regression model shows P‐values of 0.003 for OS and <0.001 PFS, confirming the prognostic value of the model score independent of MYC‐R. For the remaining analyses, including those for treatment and trial, we observed significant P‐values for PFS and OS. In contrast, if COO is included as an additional variable, the model score was rendered insignificant. This, however, might be attributable to the comparable low number of available COO labels (*N* = 449). To further evaluate the prognostic value of the model score, we used LRTs by fitting a full model that included the variable of interest and an adjusted reduced model that excluded that variable (Supporting Information S1: Table 6). Consistent with previous findings, the model score showed significant prognostic improvement, except for OS in the clinical test cohort. Our model score is a continuous variable and does not dichotomize patients like FISH for MYC‐R/DH/TH. To analyze the score in a dichotomized fashion, the external test cohort and the clinical test cohort (for baseline characteristics, see Supporting Information S1: Table 8) were reconciled and systematically tested for an association between dichotomized model scores on the one hand and OS and PFS on the other hand. Significance levels were calculated using log‐rank tests. The threshold yielding the strongest separation within the external test cohort between high‐ and low‐risk groups was selected and subsequently applied to stratify the clinical test cohort into high‐ and low‐risk groups. This optimization of patient stratification into risk groups identifies a high‐risk group with a median PFS of 47.6 months (44.7% 5‐year PFS) and a median PFS that has not been reached (77.3% 5‐year PFS) in the low‐risk group (Figure 3). Similarly, for OS, we observed a 5‐year OS rate of 54.3% for the high‐risk patients and 82.4% for the low‐risk patients (Figure 4).

### Molecular features associated with “MYCness”

Given the strong association between the model score and survival in lymphomas even without MYC‐R, we aimed to evaluate the biological context of the score. All model scores (MYC‐R or DH/TH) were significantly associated with the presence of the DH/dark zone signature (coef = 0.286, P < 0.001).^47^ A positive association was detected with the ABC COO subtype (coef = 0.138, P = 0.003) and vice versa for the GCB subtype. For 509 cases in the external and clinical cohorts, DHIT Nanostring gene expression data for 90 genes were available (Supporting Information S1: Table 9). For data preprocessing, a pseudo count was added and profiles were normalized to an average count of 1000. Finally, data were log_2_‐transformed. In addition, preprocessed gene expression for the lymphoma‐associated macrophage interaction signature (LAMIS)^48^ for an additional 269 cases was used (Supporting Information S1: Table 10). A positive correlation was found between model scores and MYC and BCL2. Moreover, the model score was inversely correlated with the expression of genes reflecting macrophages, for example, CD274, CD14, and CD68, in line with our previous data demonstrating that a high macrophage content in the tumor microenvironment is associated with a favorable outcome.^49^ Moreover, genes reflecting the microenvironment, such as the CD30 ligand (TNFSF8), were also associated with the model score. Thus, despite the fact that our score was trained to identify cases with MYC‐R, it reflects multiple biological features of neoplastic cells, including MYC expression, the DH signature, and the tumor microenvironment.

## DISCUSSION

DLBCL/HGBCL are the most common group of lymphomas in adults. Subtypes of DLBCL/HGBCL such as the COO subtypes or MYC‐R require molecular testing to be identified. Clinically relevant subtypes are DLBCL/HGBCL with MYC‐R, especially DH/TH, as they show an unfavorable outcome under standard immunochemotherapy^3^ and are thus recognized either as distinct prognostic subgroups or as entities by current classifications.^1^, ^4^ Even specialized pathologists are unable to recognize MYC‐R lymphoma using H&E slides.^2^, ^50^ Auxiliary techniques such as immunohistochemistry for MYC protein were not helpful under diagnostic conditions.^51^ Thus, current diagnostic guidelines recommend screening all lymphomas with the morphology of DLBCL/HGBCL for MYC‐R by FISH or other suitable molecular tests.^5^ However, the analysis of MYC‐R/DH/TH by FISH has several disadvantages in risk stratification of patients with DLBCL/HGBCL. First, FISH may miss a subset of lymphomas with MYC‐R.^9^, ^52^ Second, although FISH is a standard diagnostic procedure in many laboratories, access may be limited in low‐ and middle‐income countries.^53^, ^54^ Third, and most importantly, molecular detection of MYC‐R is only demarcating a small subgroup of <10% of all patients with DLBCL/HGBCL, and a considerable number of patients with high risk of relapse and unfavorable outcome remain undetected within the group of MYC‐negative lymphomas.^55^, ^56^ To further dissect MYC‐negative lymphomas into prognostic subgroups, gene expression profiling or next‐generation sequencing is required.^47^, ^55^ Here, we demonstrate that application of AI to H&E‐stained slides may help to overcome some of the aforementioned limitations of molecular profiling at least with respect to risk assessment.

We first asked whether the prediction of MYC‐R by AI using H&E WSI can be improved compared to previous studies^23^, ^24^, ^25^, ^26^, ^27^ to a level allowing substitution of molecular testing. However, although we overcame several of the technical limitations of previous studies by generating the largest data set of WSI of DLBCL/HGBCL (*n* > 2000) currently available worldwide, testing multiple state‐of‐the‐art MIL methods and automated restriction to lymphoma‐containing areas of the WSI, AI‐predicted MYC‐R/DH/TH still suffers from moderate to low specificity, as many lymphomas without MYC‐R/DH/TH receive high model scores of “MYCness.” Utilizing the model to select for specimen requiring FISH testing to confirm MYC‐R/DH/TH in a diagnostic setting would reduce the number of tests to be conducted by only about 34% compared to the current standard approach of testing all DLBCL/HGBCL.

Our second aim was to understand if AI‐based MYC prediction is capturing a common biology and clinical behavior in all DLBCL/HGBCL independent of MYC‐R, as has been observed in gene expression profiling studies.^9^ Previous studies indicated that MYC‐R/DH/TH DLBCL/HGBCL share gene expression features with a group of lymphomas lacking these genetic aberrations but showing the same poor prognosis.^9^ Assuming that a common gene expression profile will be reflected in lymphoma morphology assessed by AI in the H&E WSI, we reasoned that our AI‐based score will associate with outcome not despite but precisely because it identifies morphological features associated with MYC/DH/TH biology (“MYCness”) in lymphomas lacking genetic MYC‐R. It is noteworthy that AI‐based “MYCness” does refer to morphological features detected and quantified by our AI approach, but not necessarily to biological MYC signaling. In line with our hypothesis, the AI‐based model is in fact a better prognostic marker than the template MYC‐R that it was trained on. We conclude that our AI‐based “MYCness” score is a stronger predictor of outcome than the MYC status on which it was trained. The model retained prognostic significance independently of established clinical risk factors, such as the IPI, and identified high‐risk patients with genetically MYC‐R/DH/TH‐negative lymphomas. This indicates that the model detects aggressive disease biology rather than genetic status alone.

The features selected by the model to identify “MYCness” remain elusive as an inherent feature of AI‐based methods. However, the attention of the model to individual tiles of the WSI can be quantified and illustrated in attention maps. Visual inspection of attention maps indicates that most models that we applied assigned high attention to areas not considered to contain lymphoma tissue. Although necrosis may not represent lymphoma morphology, it may nevertheless be used by pathologists to identify lymphoma subtypes.^57^ However, areas with improper staining or non‐infiltrated tissue received high attention too. Thus, we deployed the expertise of pathologists and trained a tumor classifier that automatically excluded non‐lymphoma tissue in the training process. This procedure also ensures that the model learns from tissue regions that pathologists consider relevant for diagnosis and reduces potential shortcomings due to learning from artifacts. As a result, the attention maps become histologically plausible by utilizing regions that the pathologists themselves would focus on in visual inspection. Although this workflow, surprisingly, does not improve the performance of the model, we believe that it improves the comprehensibility of the result and increases acceptance among pathologists, which will be crucial for future clinical applications.

As discussed above, the fact that our model was trained to identify MYC‐R but also provides prognostic information in lymphomas lacking MYC‐R does not essentially come as a surprise. In fact, the gene expression‐based DH signature was developed similarly.^9^ DLBCL/HGBCL with MYC‐R/DH/TH obviously show features in their H&E appearance that are associated with an aggressive clinical course independent of the molecular genetic background,^55^ and these features seem to be captured by our model. Being trained to detect MYC‐R/DH/TH, the association with the DH/dark zone gene expression signature^9^, ^10^ as well as protein expression of MYC and BCL2^58^, ^59^ with the “MYCness” seems plausible. It is important to stress that “MYCness” in our study reflects a morphology‐based score generated by AI on H&E slides, but not a score for biological activity of MYC, although both might be related. It is noteworthy that the model result is also associated with microenvironmental signatures known to provide molecular genetic subtype‐overarching prognostic information, which have not yet been linked to MYC‐R.^49^, ^60^ Future studies will be required to identify the molecular features associated best with the model score and to understand if the training is reflecting more than just “MYCness” in the narrow sense. These future studies may also help to understand why training for MYC‐R provided a better outcome predictor compared to training for DH/TH, although the latter were the core cohort to define the DH signature by gene expression profiling and are considered a molecularly rather homogeneous entity.^9^, ^61^

Training to detect MYC‐R may appear as an indirect way to generate an outcome predictor by AI, raising the question of why AI was not trained directly to predict clinical outcome. However, robust outcome‐trained models from WSI require not only sufficiently large cohorts with clinical follow‐up but also adequate numbers of outcome events, endpoint quality, cohort diversity, treatment heterogeneity, and adequate follow‐up duration. Although our cohort of almost 1000 clinically annotated DLBCL/HGBCL cases is substantial, we considered it insufficient for reliable independent training and validation of a dedicated outcome‐prediction model within the scope of the present study. Therefore, we took advantage of the fact that large cohorts of DLBCL/HGBCL with MYC‐R/DH/TH are available from diagnostic testing and that the association of these features with outcome is well established. Nevertheless, joining WSI of DLBCL/HGBCL from multiple groups and consortia in the future is certainly desirable and may enable researchers to train AI directly for outcome prediction. So far, it remains uncertain whether such an approach will provide a better model than the one based on MYC presented in the current study. A limitation of our model as well as that of most published studies of this kind is a lack of knowledge on slide/tissue/scan features on the performance of the model. Recent studies suggest that foundation models are still susceptible to batch effects despite having diverse training data.^62^ However, the biopsies in our study were retrieved from pathology centers all over Germany, reflect the diagnostic conditions in this area, and demonstrate that the model is successful despite the heterogeneity of the tissue processing. Nevertheless, future studies should evaluate systematically the influence of tissue processing, cutting, staining, and scanning on model performance. In addition, potential benefits of color normalization and stain augmentation should be studied. The presented study showed that predictions were average when multiple slides per patient were available. Performance could be further improved with alternative aggregation strategies, such as focusing on high‐quality slides assessed by pathologists.

Our model is certainly useful in a retrospective analysis to study risk profiles and clinical outcome over multiple independent cohorts without the requirement of additional molecular testing. Once the model's performance is confirmed in additional independent cohorts, it can be used in prospective clinical trials. The “MYCness” model may be clinically useful when targeting MYC^63^ or when considering intensification of therapy for high‐risk patients.^64^ These potential use cases require the definition of cutoffs based on the purpose of the risk stratification.

In summary, we present the first morphology‐based AI model that captures “MYCness” as an aggressive phenotype of DLBCL/HGBCL from routine H&E‐stained WSIs. The predicted score provides prognostic information independent of the IPI, enabling large‐scale risk stratification in prospective clinical trials for pathologists and researchers worldwide without the need for expert knowledge in the field.

## AUTHOR CONTRIBUTIONS

**Jonas Lippl**: Conceptualization; writing—original draft; formal analysis; methodology; visualization; writing—review and editing; software. **Sarah Reinke**: Conceptualization; writing—review and editing. **Stefan Schrod**: Formal analysis; writing—review and editing. **Lukas Wolfseher**: Formal analysis; methodology; writing—review and editing. **Paul Hüttl**: Formal analysis; methodology; writing—review and editing. **Michael Huttner**: Formal analysis; methodology; writing—review and editing. **Annette M. Staiger**: Writing—review and editing; data curation. **Ilske Oschlies**: Writing—review and editing; data curation. **Karoline Koch**: Writing—review and editing; data curation. **Edward G. Michaelis**: Writing—review and editing; data curation. **Julia Richter**: Writing—review and editing; data curation. **Hilka Rauert‐Wunderlich**: Writing—review and editing; data curation. **Nicole Seifert**: Formal analysis; methodology; writing—review and editing. **Robin Kosch**: Formal analysis; methodology; writing—review and editing. **Norbert Schmitz**: Writing—review and editing; data curation. **Marita Ziepert**: Writing—review and editing; data curation. **Lorenz Thurner**: Writing—review and editing; data curation. **Viola Poeschel**: Writing—review and editing; data curation. **Bertram Glaß**: Writing—review and editing; data curation. **Lorenz Trümper**: Writing—review and editing; data curation. **Gerhard Held**: Writing—review and editing; data curation. **David W. Scott**: Writing—review and editing; data curation. **Andreas Rosenwald**: Writing—review and editing; data curation; funding acquisition. **German Ott**: Writing—review and editing; data curation; funding acquisition. **Rainer Spang**: Writing—review and editing; conceptualization; funding acquisition. **Michael Altenbuchinger**: Writing—review and editing; conceptualization; writing—original draft; supervision; funding acquisition. **Wolfram Klapper**: Conceptualization; writing—review and editing; writing—original draft; data curation; funding acquisition.

## CONFLICT OF INTEREST STATEMENT

H.R.‐W.: Speaker for Incyte Corporation. A.R.: Institutional research contract—MorphoSys and Incyte. S.R.: Institutional research funding from Incyte. W.K.: Institutional research funding from Incyte, Amgen, Roche, and Janssen. The other authors report no competing interests.

## ETHICS STATEMENT

This study was conducted according to the recommendations of the Institutional Review Board of the Medical Faculty, University of Kiel (number D447/10), and is covered by the patient‐informed consent of the respective trials included.

## FUNDING

This work was supported by the German Federal Ministry of Education and Research (BMBF) as project FDLP under grant numbers 01KD2209A, 01KD2209B, 01KD2209C, 01KD2209D, and 01KD2209E, and project FDLP2 under grant numbers 01KD2415A, 01KD2415B, 01KD2415C, 01KD2415D, and 01KD2415E. Additional support was obtained from the Lunenburg Lymphoma Biomarker Consortium (LLBC). G.O. was additionally supported by the Robert‐Bosch‐Foundation, Stuttgart. Open Access funding enabled and organized by Projekt DEAL.

## Supporting information

Supporting Information.

Supporting Information.

## Data Availability

The data that support the findings of this study are available on request from the corresponding author. The data are not publicly available due to privacy or ethical restrictions.
