# Supplementary material for: AI for prognostic assessment of diffuse large B‐cell lymphoma using H&E whole‐slide images
Source: Hemasphere. 2026 Aug 31;10(9):e70463. doi: 10.1002/hem3.70463 (PMC13528628; doi:10.1002/hem3.70463)
Supplement: Supplementary file 2 [file HEM3-10-e70463-s001.docx]

**Supplementary Methods**

**Whole Slide Imaging**

All samples in the development cohort were scanned using a Ventana DP 200 scanner at a resolution of 0.25 microns per pixel (mpp). For the external test cohort, the NanoZoomer S60 was mainly used, with a resolution of 0.221 mpp. Some cases were scanned using the NanoZoomer 2.0 RS, with a resolution of 0.227 mpp, and the aforementioned Ventana DP 200.

**Tissue Classification**

For our tissue classifier, expert pathologists manually labeled nearly 28,000 DLBCL patches from 67 WSIs. To make the labeling process more efficient, we implemented a tool called Mechanical Turk (https://turk.spang-lab.de/pamly) that uses Pamly^1^ to display similar patches in a 6 x 9 grid. Pathologists can select a tissue label for each set of images and then remove any patches that do not fit from the grid. The selected label is then applied to the entire set of patches, allowing for quick labeling. We generated UNI^2^ embeddings for the labeled patches and trained a classifier using stochastic gradient descent (SGD) with an initial learning rate of 0.001, followed by learning rate decay, to predict a binary tumor/non-tumor label. Of all the WSIs, 46 were used for training, 10 for validation, and 11 for testing. Test accuracy was 0.875, and the test area under the curve (AUC) was 0.921. Tumor tissue accounted for approximately 66% of all patches. To include only patches in which the model had high confidence that the tissue was tumor tissue, we included only tumor patches with a predicted tumor probability of at least 80%.

**MIL Methods**

This section provides more technical details about the six MIL methods used and the training process. These methods differ in how they aggregate patch-level information, incorporate spatial context, or in their architectural structure. Below, we briefly summarize the key characteristics of each method used in our study:

ABMIL^3^ where the first to propose using the attention mechanism^4,5^ to obtain a weighted average of the instance embeddings in the bag, where the weights are determined by a neural network.

CLAM^6^ uses attention-based pooling similar to ABMIL, however, they use multiple branches in parallel, allowing each of them to accumulate class specific features. In addition, they propose instance level clustering to further refine the feature space. In addition to the multi-branch version of CLAM (CLAM-MB), a single-branch version (CLAM-SB) is available. However, this version was not used in this manuscript.^6^

DTFD-MIL^7^ randomly splits the embeddings into pseudo-bags to artificially increase the number of bags. On those pseudo-bags a first MIL model is trained to aggregate them, and a second MIL model is trained on the aggregated embeddings from each pseudo bag to obtain a final slide prediction.^7^

AttriMIL^8^ uses an additional instance-level classifier to assign a prediction to each instance. Each instance prediction is multiplied by its attention score to obtain a new attribution score. Summing up the attribution scores across classes yields the final prediction.^8^

Previous methods treat instances as independent and identically distributed. However, these methods neglect spatial information and correlations between instances. Therefore, TransMIL^9^ proposes a transformer-based MIL method that uses the self-attention mechanism to exploit morphological and spatial information for the pairwise correlation between the tiles in an image.^9^

Similarly, Snuffy^10^ uses sparse transformers, where each attention head computes attention with only a subset of features to increase computational efficiency.^10^

We trained each model in one iteration of 5-fold cross-validation for each setting. Then, we used the five models as an ensemble, averaging the predicted probabilities to obtain predictions for the test sets. It would be possible to increase the number of iterations to get more models for the ensemble and get a more accurate estimation of cross-validation performance. However, we observed that increasing the number of iterations does not significantly improve test performance. Due to the large number of configurations in the experiments increasing the iterations would also render the time required for the experiments impractical. We trained all models distributed on eight NVIDIA A100 GPUs with a per-GPU batch-size of 1, i.e. an effective batch-size of 8, for a maximum of 50 epochs. The random seed was set to 42 for reproducibility. The initial learning rate was set to ƞ=10^-4^ and a cosine scheduler for learning rate decay after linear warm up of 5 epochs was used. The AdamW^11^ optimizer, with weight decay 0.01, was used for all runs, and early stopping with a patience of 10 was employed when validation loss was minimal. The full training and analysis code is provided at https://gitlab.gwdg.de/jlipl/dlbcl.

**Supplementary Data**

**Supplementary Fig. 1: Evaluation of ROC curves for MYC-R predictions.**

Figure 1

ROC curves for the cross-validation of the development cohort (a, *N*=1035), and external test cohort (b, *N*=499) for the MYC-R classification task. The blue lines indicate the predictions from the single models obtained by the five cross-validation folds. The red line is the average prediction of the ensemble of models obtained from cross-validation. The dashed diagonal line in the plots represents an AUC of 0.5, which is achieved by a random guessing model.

**Supplementary Fig. 2: Evaluation of confusion matrices for MYC-R predictions.** Confusion matrices for the cross-validation split of the development cohort (a), and external test cohort (b) for the model trained to detect MYC-R. A threshold of 0.30 for the MYC-R score was selected using the maximum balanced accuracy of the cross-validation predictions on the development cohort.

**Supplementary Figure 3: Calibration curves for MYC-R predictions in cross-validation (a) and for the external test cohort (b).** Dashed lines indicate perfect calibration, and blue markers show observed event frequencies across predicted-probability bins. Reported metrics for calibration include Brier-score and Expected Calibration Error (ECE).

**Supplementary Fig. 4: Kaplan-Meier Curves stratified by FISH annotations.** Kaplan-Meier curves for OS (a & c) and PFS (b & d) for the Clinical Test Cohort. Patients were stratified according to their MYC-R (a & b) and DH/TH (c & d) FISH labels.

**Supplementary Fig. 5: Stratification of patients within MYC- and MYC+ subgroups.** The optimal stratification threshold was applied to OS (a & c) and PFS (b & d) for patients in the external test cohort without MYC-R (MYC-, a & b) and with MYC-R (MYC+, c & d).

**Supplementary Table 1: Distribution of Development and Test Cohorts by MYC/DH/TH Status and Availability of FISH and Clinical Annotations.** Shown is the distribution of samples across the development cohort, including the number of MYC- and DH/TH-positive cases. For the seven clinical trials, the number of patients with FISH annotations and those with available clinical data is reported. In addition, the total number of patients enrolled in the respective trials is reported. Patients with FISH annotations were assigned to the external test cohort (*N*=499), whereas patients without FISH data were included in the clinical test cohort (*N*=484). For a subset of patients, LAMIS and DHIT gene expression data, double hit/dark zone signature classification, and the COO subtype are available.

| Dataset | Patients with FISH-Annotation | MYC+ Patients | DH/TH Patients | Patients without FISH-Annotation | Patients with Clinical Data | Total Published Number of Patients | LAMIS Gene Expression Data | DHIT Gene Expression Data | Double Hit/Dark Zone Signature | COO Subtype | Treatment, n (%) | IPI group, n (%) |
| --- | --- | --- | --- | --- | --- | --- | --- | --- | --- | --- | --- | --- |
| Development | 1035 | 334 | 188 | 0 | 0 | - | - | - | 0 | 0 | - | - |
| Denser | 45 | 3 | 1 | 45 | 90 | 124 | 55 | 46 | 44 | 39 | 6 x CHOP-14 + 12xR: 90 (100.0%) | low: 25 (27.8%);  low-intermediate: 18 (20.0%);  high-intermediate: 28 (31.1%);  high: 19 (21.1%) |
| Rivover-60 | 97 | 13 | 8 | 25 | 122 | 1222 | 44 | 44 | 40 | 36 | 6 x CHOP-14 + 8xR: 35 (28.7%);  8 x CHOP-14: 32 (26.2%);  6 x CHOP-14: 28 (23.0%);  8 x CHOP-14 + 8xR: 27 (22.1%) | low: 45 (36.9%);  low-intermediate: 33 (27.0%);  high-intermediate: 26 (21.3%);  high: 18 (14.8%) |
| Ricover-noRTh | 69 | 5 | 2 | 46 | 115 | 166 | 73 | 63 | 60 | 57 | 6 x CHOP-14 + 8xR: 115 (100.0%) | low: 30 (26.1%);  low-intermediate: 31 (27.0%);  high-intermediate: 33 (28.7%);  high: 21 (18.3%) |
| SMARTER | 74 | 11 | 7 | 66 | 141 | 189 | 85 | 77 | 71 | 69 | 6 x CHOP-14 + 12xR: 140 (100.0%) | low: 41 (29.3%); low-intermediate: 35 (25.0%); high-intermediate: 39 (27.9%);  high: 25 (17.9%) |
| MINT | 74 | 3 | 0 | 29 | 103 | 824 | 0 | 3 | 25 | 19 | 6 x CHOEP-21 + 6xR: 54 (52.4%);  6 x CHOEP-21: 49 (47.6%) | low: 93 (90.3%);  low-intermediate: 9 (8.7%);  high-intermediate: 1 (1.0%);  high: 0 (0.0%) |
| MegaCHOEP | 13 | 1 | 1 | 15 | 28 | 77 | 12 | 12 | 12 | 10 | MegaCHOEP-21 + 6xR: 14 (50.0%);  8 x CHOEP-14 + 6xR: 14 (50.0%) | low: 0 (0.0%);  low-intermediate: 16 (57.1%);  high-intermediate: 8 (28.6%);  high: 4 (14.3%) |
| Unfolder | 127 | 8 | 5 | 258 | 385 | 695 | 0 | 264 | 228 | 219 | 6 x R-CHOP-21: 109 (28.3%);  6 x R-CHOP-14 + RTh: 96 (24.9%);  6 x R-CHOP-14: 96 (24.9%);  6 x R-CHOP-21 + RTh: 84 (21.8%) | low: 316 (82.1%);  low-intermediate: 66 (17.1%);  high-intermediate: 3 (0.8%);  high: 0 (0.0%) |
| Total | 499 | 44 | 24 | 484 | 983 | 3297 | 269 | 509 | 480 | 449 | - | low: 550 (56.0%);  low-intermediate: 208 (21.2%);  high-intermediate: 138 (14.0%);  high: 87 (8.9%) |

**Supplementary Table 2: MYC-classification results for the six tested methods with and without the tumor classifier prefiltering step.** Reported metrics are the ROC AUC and the AUPRC for cross-validation, and the external test cohort.

| **Method** | **Tumor Only** | **Cross-Val AUC** | **Test AUC (External)** | **Test AUPRC (External)** |
| --- | --- | --- | --- | --- |
| ABMIL | 🗶 | 0.728 ± 0.060 | 0.736 | 0.256 |
| ABMIL | ✓ | 0.698 ± 0.050 | 0.743 | 0.285 |
| CLAM | 🗶 | **0.824 ± 0.036** | 0.804 | **0.480** |
| CLAM | ✓ | 0.811 ± 0.035 | 0.774 | 0.406 |
| AttriMIL | 🗶 | 0.814 ± 0.025 | 0.800 | 0.403 |
| AttriMIL | ✓ | 0.810 ± 0.038 | 0.745 | 0.289 |
| DTFD-MIL | 🗶 | 0.814 ± 0.020 | 0.790 | 0.428 |
| DTFD-MIL | ✓ | 0.815 ± 0.032 | 0.771 | 0.371 |
| Snuffy | 🗶 | 0.744 ± 0.051 | 0.679 | 0.154 |
| Snuffy | ✓ | 0.770 ± 0.019 | 0.624 | 0.145 |
| TransMIL | 🗶 | 0.812 ± 0.025 | **0.825** | 0.460 |
| TransMIL | ✓ | 0.796 ± 0.038 | 0.791 | 0.378 |

**Supplementary Table 3: Performance of the six tested methods for DH/TH-classification with and without tumor classifier prefiltering.** Classification results are summarized using ROC AUC and AUPRC across cross-validation, and the external test cohort.

| **Method** | **Tumor Only** | **Cross-Val AUC** | **Test AUC (External)** | **Test AUPRC (External)** |
| --- | --- | --- | --- | --- |
| ABMIL | 🗶 | 0.742 ± 0.071 | 0.732 | 0.112 |
| ABMIL | ✓ | 0.712 ± 0.044 | 0.694 | **0.232** |
| CLAM | 🗶 | **0.836 ± 0.021** | **0.778** | 0.186 |
| CLAM | ✓ | 0.817 ± 0.034 | 0.748 | 0.151 |
| AttriMIL | 🗶 | 0.817 ± 0.014 | 0.765 | 0.142 |
| AttriMIL | ✓ | 0.811 ± 0.047 | 0.653 | 0.082 |
| DTFD-MIL | 🗶 | 0.816 ± 0.019 | 0.743 | 0.143 |
| DTFD-MIL | ✓ | 0.813 ± 0.032 | 0.736 | 0.125 |
| Snuffy | 🗶 | 0.761 ± 0.034 | 0.583 | 0.069 |
| Snuffy | ✓ | 0.767 ± 0.060 | 0.558 | 0.066 |
| TransMIL | 🗶 | 0.820 ± 0.011 | 0.766 | 0.185 |
| TransMIL | ✓ | 0.803 ± 0.034 | 0.711 | 0.164 |

**Supplementary Table 4: Effectiveness of the six methods examined for MYC/BCL2-classification with and without tumor classifier prefiltering.** The summary of classification results uses ROC AUC and AUPRC across cross-validation and the external test cohort.

| **Method** | **Tumor Only** | **Cross-Val AUC** | **Test AUC (External)** | **Test AUPRC (External)** |
| --- | --- | --- | --- | --- |
| ABMIL | 🗶 | 0.713 ± 0.072 | 0.759 | 0.088 |
| ABMIL | ✓ | 0.698 ± 0.050 | 0.807 | 0.157 |
| CLAM | 🗶 | **0.824 ± 0.036** | 0.852 | 0.284 |
| CLAM | ✓ | 0.811 ± 0.035 | 0.814 | 0.237 |
| AttriMIL | 🗶 | 0.814 ± 0.025 | 0.852 | 0.252 |
| AttriMIL | ✓ | 0.810 ± 0.038 | 0.785 | 0.172 |
| DTFD-MIL | 🗶 | 0.818 ± 0.018 | 0.843 | 0.267 |
| DTFD-MIL | ✓ | 0.815 ± 0.031 | 0.813 | 0.226 |
| Snuffy | 🗶 | 0.744 ± 0.049 | 0.657 | 0.066 |
| Snuffy | ✓ | 0.770 ± 0.029 | 0.658 | 0.061 |
| TransMIL | 🗶 | 0.811 ± 0.025 | 0.859 | 0.224 |
| TransMIL | ✓ | 0.796 ± 0.038 | 0.830 | 0.221 |

**Supplementary Table 5: Cox regression analysis for PFS and OS across external and clinical test cohorts.** Hazard ratios (HR=exp(coef)) and their corresponding coefficients (coef), standard errors (se), 95% confidence intervals (CI), and *P*-values are reported for each covariate. The first two rows describe a Cox regression analysis that uses only model scores (probabilities between 0 and 1). The remaining rows include predicted model scores, IPI risk groups, and MYC FISH labels in the analysis. Additionally, further Cox regression analyses are included with the cohort and treatment as covariates. Statistically significant results are indicated by a *P*-value of less than 0.05.

|  |  | **External Test Cohort** | | | | | | | | | **Clinical Test Cohort** | | | | | | | | | | |
| --- | --- | --- | --- | --- | --- | --- | --- | --- | --- | --- | --- | --- | --- | --- | --- | --- | --- | --- | --- | --- | --- |
| **Time** | **covariate** | **coef** | **exp(coef)** | **se(coef)** | **coef lower 95% CI** | **coef upper 95% CI** | **exp(coef) lower 95% CI** | **exp(coef) upper 95% CI** | **z** | ***P*-value** | **coef** | **exp(coef)** | **se(coef)** | **coef lower 95% CI** | | **coef upper 95% CI** | **exp(coef) lower 95% CI** | | **exp(coef) upper 95% CI** | **z** | ***P*-value** |
| PFS | model score | 2.229 | 9.293 | 0.504 | 1.241 | 3.217 | 3.459 | 24.964 | 4.421 | **<0.001** | 1.762 | 5.822 | 0.482 | 0.817 | 2.706 | | | 2.265 | 14.966 | 3.657 | **<0.001** |
| OS | model score | 2.270 | 9.684 | 0.583 | 1.128 | 3.413 | 3.089 | 30.361 | 3.894 | **<0.001** | 1.711 | 5.537 | 0.579 | 0.577 | 2.846 | | | 1.781 | 17.220 | 2.957 | **0.003** |
| PFS | MYC | 0.486 | 1.626 | 0.285 | -0.073 | 1.045 | 0.930 | 2.843 | 1.705 | 0.088 | - | - | - | - | - | | | - | - | - | - |
| OS | MYC | 0.536 | 1.710 | 0.323 | -0.096 | 1.169 | 0.908 | 3.220 | 1.661 | 0.097 | - | - | - | - | - | | | - | - | - | - |
| PFS | model score | 2.203 | 9.050 | 0.542 | 1.140 | 3.265 | 3.128 | 26.182 | 4.064 | **<0.001** | - | - | - | - | - | | | - | - | - | - |
|  | MYC | 0.041 | 1.042 | 0.307 | -0.561 | 0.643 | 0.571 | 1.902 | 0.133 | 0.894 | - | - | - | - | - | | | - | - | - | - |
| OS | model score | 2.222 | 9.229 | 0.631 | 0.985 | 3.460 | 2.678 | 31.812 | 3.52 | **<0.001** | - | - | - | - | - | | | - | - | - | - |
|  | MYC | 0.069 | 1.072 | 0.351 | -0.619 | 0.757 | 0.539 | 2.133 | 0.198 | 0.843 | - | - | - | - | - | | | - | - | - | - |
| PFS | model score | 2.017 | 7.515 | 0.544 | 0.951 | 3.083 | 2.587 | 21.824 | 3.708 | **<0.001** | 1.516 | 4.555 | 0.492 | 0.552 | 2.480 | | | 1.737 | 11.943 | 3.083 | **0.002** |
|  | IPI low-intermediate | 0.543 | 1.721 | 0.257 | 0.039 | 1.046 | 1.040 | 2.847 | 2.112 | **0.035** | 0.312 | 1.367 | 0.258 | -0.193 | 0.817 | | | 0.825 | 2.264 | 1.212 | 0.225 |
|  | IPI high-intermediate | 1.145 | 3.144 | 0.245 | 0.665 | 1.626 | 1.944 | 5.083 | 4.673 | **<0.001** | 1.085 | 2.960 | 0.253 | 0.589 | 1.582 | | | 1.802 | 4.863 | 4.285 | **<0.001** |
|  | IPI high | 1.533 | 4.630 | 0.279 | 0.987 | 2.079 | 2.682 | 7.993 | 5.501 | **<0.001** | 1.370 | 3.936 | 0.276 | 0.829 | 1.912 | | | 2.291 | 6.763 | 4.962 | **<0.001** |
|  | MYC | -0.173 | 0.841 | 0.310 | -0.781 | 0.434 | 0.458 | 1.544 | -0.559 | 0.576 |  |  |  |  |  | | |  |  |  |  |
| OS | model score | 2.014 | 7.496 | 0.636 | 0.769 | 3.26 | 2.157 | 26.055 | 3.169 | **0.002** | 1.343 | 3.832 | 0.591 | 0.186 | 2.501 | | | 1.204 | 12.193 | 2.275 | **0.023** |
|  | IPI low-intermediate | 0.468 | 1.597 | 0.316 | -0.152 | 1.088 | 0.859 | 2.968 | 1.479 | 0.139 | 0.574 | 1.775 | 0.316 | -0.045 | 1.193 | | | 0.956 | 3.297 | 1.817 | 0.069 |
|  | IPI high-intermediate | 1.420 | 4.137 | 0.278 | 0.876 | 1.964 | 2.401 | 7.128 | 5.115 | **<0.001** | 1.360 | 3.894 | 0.308 | 0.755 | 1.964 | | | 2.129 | 7.125 | 4.411 | **<0.001** |
|  | IPI high | 1.680 | 5.364 | 0.319 | 1.054 | 2.306 | 2.869 | 10.03 | 5.261 | **<0.001** | 1.761 | 5.821 | 0.317 | 1.140 | 2.382 | | | 3.128 | 10.832 | 5.559 | **<0.001** |
|  | MYC | -0.139 | 0.87 | 0.354 | -0.832 | 0.554 | 0.435 | 1.741 | -0.393 | 0.694 |  |  |  |  |  | | |  |  |  |  |
| PFS | model score | 1.634 | 5.125 | 0.513 | 0.629 | 2.639 | 1.875 | 14.005 | 3.186 | **0.001** | 1.329 | 3.778 | 0.504 | 0.342 | 2.316 | | | 1.408 | 10.137 | 2.639 | **0.008** |
|  | MINT | -0.525 | 0.592 | 0.389 | -1.288 | 0.238 | 0.276 | 1.269 | -1.349 | 0.177 | -0.811 | 0.444 | 0.443 | -1.679 | 0.056 | | | 0.187 | 1.057 | -1.834 | 0.067 |
|  | MegaCHOEP | 0.231 | 1.260 | 0.529 | -0.805 | 1.267 | 0.447 | 3.55 | 0.437 | 0.662 | -0.143 | 0.867 | 0.499 | -1.121 | 0.835 | | | 0.326 | 2.306 | -0.286 | 0.775 |
|  | RICOVER-noRTh | 0.164 | 1.179 | 0.366 | -0.552 | 0.881 | 0.576 | 2.414 | 0.45 | 0.653 | -0.544 | 0.581 | 0.353 | -1.236 | 0.149 | | | 0.290 | 1.160 | -1.539 | 0.124 |
|  | RICOVER60 | 0.618 | 1.856 | 0.322 | -0.012 | 1.249 | 0.988 | 3.487 | 1.922 | 0.055 | -0.395 | 0.674 | 0.437 | -1.252 | 0.462 | | | 0.286 | 1.587 | -0.904 | 0.366 |
|  | SMARTER | -0.357 | 0.700 | 0.403 | -1.148 | 0.434 | 0.317 | 1.543 | -0.885 | 0.376 | -0.185 | 0.831 | 0.308 | -0.789 | 0.419 | | | 0.454 | 1.520 | -0.601 | 0.548 |
|  | Unfolder | -1.191 | 0.304 | 0.396 | -1.967 | -0.416 | 0.140 | 0.660 | -3.011 | **0.003** | -1.354 | 0.258 | 0.281 | -1.904 | -0.803 | | | 0.149 | 0.448 | -4.820 | **<0.001** |
| OS | model score | 1.601 | 4.959 | 0.591 | 0.442 | 2.76 | 1.556 | 15.804 | 2.708 | **0.007** | 0.968 | 2.633 | 0.609 | -0.225 | 2.162 | | | 0.798 | 8.686 | 1.590 | 0.112 |
|  | MINT | -1.110 | 0.329 | 0.481 | -2.052 | -0.168 | 0.128 | 0.845 | -2.311 | 0.021 | -0.948 | 0.387 | 0.476 | -1.881 | -0.015 | | | 0.152 | 0.985 | -1.992 | **0.046** |
|  | MegaCHOEP | 0.201 | 1.223 | 0.587 | -0.949 | 1.351 | 0.387 | 3.863 | 0.343 | 0.731 | -0.216 | 0.805 | 0.552 | -1.298 | 0.866 | | | 0.273 | 2.377 | -0.392 | 0.695 |
|  | RICOVER-noRTh | 0.253 | 1.288 | 0.395 | -0.520 | 1.027 | 0.594 | 2.792 | 0.642 | 0.521 | -0.688 | 0.503 | 0.391 | -1.455 | 0.079 | | | 0.233 | 1.082 | -1.758 | 0.079 |
|  | RICOVER60 | 0.570 | 1.769 | 0.352 | -0.120 | 1.260 | 0.887 | 3.526 | 1.619 | 0.105 | -0.718 | 0.488 | 0.503 | -1.704 | 0.268 | | | 0.182 | 1.307 | -1.428 | 0.153 |
|  | SMARTER | -0.667 | 0.513 | 0.488 | -1.623 | 0.289 | 0.197 | 1.335 | -1.367 | 0.172 | -0.407 | 0.665 | 0.343 | -1.080 | 0.265 | | | 0.340 | 1.303 | -1.188 | 0.235 |
|  | Unfolder | -1.510 | 0.221 | 0.467 | -2.425 | -0.595 | 0.088 | 0.552 | -3.233 | **0.001** | -2.059 | 0.128 | 0.348 | -2.741 | -1.377 | | | 0.065 | 0.252 | -5.918 | **<0.001** |
| PFS | model score | 1.620 | 5.055 | 0.511 | 0.618 | 2.623 | 1.855 | 13.775 | 3.168 | **0.002** | 1.112 | 3.042 | 0.510 | 0.113 | 2.111 | | | 1.120 | 8.260 | 2.182 | **0.029** |
|  | 6 x CHOEP-21 + 6xR | -0.556 | 0.573 | 0.518 | -1.572 | 0.459 | 0.208 | 1.583 | -1.074 | 0.283 | -1.883 | 0.152 | 1.081 | -4.001 | 0.235 | | | 0.018 | 1.265 | -1.743 | 0.081 |
|  | 6 x CHOP-14 | 1.424 | 4.153 | 0.452 | 0.538 | 2.310 | 1.713 | 10.071 | 3.151 | **0.002** | 0.892 | 2.441 | 0.712 | -0.503 | 2.288 | | | 0.605 | 9.852 | 1.253 | 0.210 |
|  | 6 x CHOP-14 + 12xR | 0.056 | 1.058 | 0.417 | -0.761 | 0.873 | 0.467 | 2.395 | 0.134 | 0.893 | 0.144 | 1.155 | 0.441 | -0.720 | 1.009 | | | 0.487 | 2.743 | 0.327 | 0.743 |
|  | 6 x CHOP-14 + 8xR | 0.469 | 1.598 | 0.414 | -0.342 | 1.280 | 0.710 | 3.597 | 1.133 | 0.257 | -0.361 | 0.697 | 0.494 | -1.329 | 0.607 | | | 0.265 | 1.834 | -0.732 | 0.464 |
|  | 6 x R-CHOP-14 | -1.813 | 0.163 | 0.791 | -3.364 | -0.262 | 0.035 | 0.769 | -2.291 | **0.022** | -1.467 | 0.231 | 0.557 | -2.558 | -0.376 | | | 0.077 | 0.687 | -2.635 | 0.008 |
|  | 6 x R-CHOP-14 + RTh | -0.297 | 0.743 | 0.614 | -1.500 | 0.907 | 0.223 | 2.477 | -0.483 | 0.629 | -1.648 | 0.192 | 0.580 | -2.786 | -0.511 | | | 0.062 | 0.600 | -2.841 | 0.005 |
|  | 6 x R-CHOP-21 | -0.885 | 0.413 | 0.571 | -2.005 | 0.235 | 0.135 | 1.264 | -1.549 | 0.121 | -0.672 | 0.511 | 0.476 | -1.604 | 0.260 | | | 0.201 | 1.297 | -1.414 | 0.157 |
|  | 6 x R-CHOP-21 + RTh | -0.853 | 0.426 | 0.677 | -2.181 | 0.475 | 0.113 | 1.607 | -1.259 | 0.208 | -1.047 | 0.351 | 0.542 | -2.110 | 0.016 | | | 0.121 | 1.016 | -1.931 | 0.054 |
|  | 8 x CHOEP-14 + 6xR | -0.515 | 0.597 | 1.066 | -2.605 | 1.574 | 0.074 | 4.826 | -0.483 | 0.629 | 0.569 | 1.766 | 0.649 | -0.704 | 1.841 | | | 0.495 | 6.305 | 0.876 | 0.381 |
|  | 8 x CHOP-14 | 0.879 | 2.410 | 0.476 | -0.054 | 1.813 | 0.947 | 6.128 | 1.847 | 0.065 | -15.803 | 0 | 1687.49 | -3323.2 | 3291.62 | | | 0 | inf | -0.009 | 0.993 |
|  | 8 x CHOP-14 + 8xR | 0.362 | 1.436 | 0.549 | -0.715 | 1.438 | 0.489 | 4.214 | 0.658 | 0.510 | 0.576 | 1.779 | 0.719 | -0.834 | 1.985 | | | 0.434 | 7.282 | 0.801 | 0.423 |
|  | MegaCHOEP-21 + 6xR | 1.011 | 2.749 | 0.618 | -0.200 | 2.223 | 0.818 | 9.232 | 1.636 | 0.102 | -0.823 | 0.439 | 1.083 | -2.944 | 1.299 | | | 0.053 | 3.667 | -0.760 | 0.447 |
| OS | model score | 1.531 | 4.624 | 0.582 | 0.390 | 2.673 | 1.477 | 14.477 | 2.629 | **0.009** | 0.710 | 2.034 | 0.616 | -0.497 | 1.917 | | | 0.609 | 6.799 | 1.153 | 0.249 |
|  | 6 x CHOEP-21 + 6xR | 0.110 | 1.116 | 0.731 | -1.323 | 1.542 | 0.266 | 4.676 | 0.150 | 0.881 | -1.641 | 0.194 | 1.096 | -3.790 | 0.507 | | | 0.023 | 1.661 | -1.497 | 0.134 |
|  | 6 x CHOP-14 | 1.973 | 7.196 | 0.672 | 0.656 | 3.291 | 1.927 | 26.869 | 2.936 | **0.003** | -0.214 | 0.807 | 1.098 | -2.366 | 1.937 | | | 0.094 | 6.940 | -0.195 | 0.845 |
|  | 6 x CHOP-14 + 12xR | 0.878 | 2.407 | 0.636 | -0.369 | 2.125 | 0.692 | 8.376 | 1.381 | 0.167 | 0.236 | 1.266 | 0.485 | -0.715 | 1.186 | | | 0.489 | 3.275 | 0.486 | 0.627 |
|  | 6 x CHOP-14 + 8xR | 1.462 | 4.315 | 0.627 | 0.233 | 2.691 | 1.263 | 14.748 | 2.332 | **0.020** | -0.300 | 0.741 | 0.547 | -1.371 | 0.772 | | | 0.254 | 2.164 | -0.548 | 0.584 |
|  | 6 x R-CHOP-14 | -1.412 | 0.244 | 1.155 | -3.676 | 0.852 | 0.025 | 2.344 | -1.222 | 0.222 | -1.707 | 0.181 | 0.671 | -3.022 | -0.391 | | | 0.049 | 0.676 | -2.542 | **0.011** |
|  | 6 x R-CHOP-14 + RTh | 0.056 | 1.058 | 0.914 | -1.735 | 1.846 | 0.176 | 6.337 | 0.061 | 0.951 | -2.454 | 0.086 | 0.839 | -4.098 | -0.809 | | | 0.017 | 0.445 | -2.924 | **0.003** |
|  | 6 x R-CHOP-21 | -0.358 | 0.699 | 0.818 | -1.961 | 1.245 | 0.141 | 3.474 | -0.437 | 0.662 | -1.230 | 0.292 | 0.576 | -2.359 | -0.101 | | | 0.095 | 0.904 | -2.135 | **0.033** |
|  | 6 x R-CHOP-21 + RTh | 0.272 | 1.313 | 0.817 | -1.33 | 1.874 | 0.265 | 6.514 | 0.333 | 0.739 | -1.561 | 0.21 | 0.681 | -2.896 | -0.225 | | | 0.055 | 0.798 | -2.291 | **0.022** |
|  | 8 x CHOEP-14 + 6xR | 0.656 | 1.927 | 1.163 | -1.624 | 2.936 | 0.197 | 18.842 | 0.564 | 0.573 | 0.611 | 1.843 | 0.736 | -0.83 | 2.053 | | | 0.436 | 7.791 | 0.831 | 0.406 |
|  | 8 x CHOP-14 | 1.949 | 7.024 | 0.672 | 0.633 | 3.266 | 1.882 | 26.205 | 2.902 | **0.004** | -16.303 | 0 | 2443.2 | -4804.9 | 4772.28 | | | 0 | inf | -0.007 | 0.995 |
|  | 8 x CHOP-14 + 8xR | 1.554 | 4.730 | 0.720 | 0.143 | 2.965 | 1.154 | 19.39 | 2.159 | **0.031** | 0.979 | 2.662 | 0.747 | -0.485 | 2.443 | | | 0.616 | 11.509 | 1.311 | 0.190 |
|  | MegaCHOEP-21 + 6xR | 1.844 | 6.320 | 0.824 | 0.228 | 3.459 | 1.256 | 31.801 | 2.236 | **0.025** | -0.462 | 0.630 | 1.100 | -2.617 | 1.693 | | | 0.073 | 5.436 | -0.420 | 0.674 |
| PFS | model score | 2.435 | 11.411 | 0.813 | 0.842 | 4.027 | 2.321 | 56.110 | 2.996 | **0.003** | 1.029 | 2.797 | 0.795 | -0.529 | 2.586 | | | 0.589 | 13.280 | 1.295 | 0.195 |
|  | GCB COO | -0.565 | 0.568 | 0.282 | -1.118 | -0.012 | 0.327 | 0.989 | -2.001 | **0.045** | -0.204 | 0.816 | 0.347 | -0.884 | 0.476 | | | 0.413 | 1.609 | -0.588 | 0.557 |
| OS | model score | 2.613 | 13.642 | 0.910 | 0.830 | 4.397 | 2.292 | 81.180 | 2.872 | **0.004** | 0.697 | 2.008 | 0.963 | -1.190 | 2.584 | | | 0.304 | 13.250 | 0.724 | 0.469 |
|  | GCB COO | -0.475 | 0.622 | 0.322 | -1.105 | 0.156 | 0.331 | 1.169 | -1.475 | 0.140 | -0.010 | 0.990 | 0.439 | -0.870 | 0.851 | | | 0.419 | 2.342 | -0.022 | 0.982 |

**Supplementary Table 6: Likelihood-ratio analysis of the external and clinical test cohorts for PFS and OS outcomes.** The table shows the likelihood-ratio test *P*-values and statistics indicating improvement in a Cox model when a variable is included compared to a model without the variable (using only the remaining variables). Large *P*-values and small LR statistics indicate significant incremental prognostic value beyond that of the other covariates.

|  |  | **External Test Cohort** | | **Clinical Test Cohort** | |
| --- | --- | --- | --- | --- | --- |
| **Time** | **Variable** | ***P-*value** | **LR-stat** | ***P-*value** | **LR-stat** |
| PFS | model score | **0.003** | 8.979 | **0.027** | 4.870 |
|  | IPI | **<0.001** | 17.355 | 0.077 | 6.856 |
|  | treatment | 0.155 | 11.920 | **0.011** | 19.918 |
|  | cohort | **<0.001** | 25.488 | **0.019** | 15.220 |
| OS | model score | **0.011** | 6.496 | 0.225 | 1.473 |
|  | IPI | **<0.001** | 17.038 | 0.104 | 6.163 |
|  | treatment | 0.753 | 5.039 | 0.127 | 12.580 |
|  | cohort | **0.020** | 15.028 | 0.058 | 12.196 |

**Supplementary Table 7: Comparison of *C*-index across different methods.** A total of six methods were evaluated for their ability to predict PFS and OS in external and Clinical Test Cohorts, with the *C*-index used as metric.

|  |  | **MYC Scores** | | | | | | | | **DH/TH Scores** | | | | | | | |
| --- | --- | --- | --- | --- | --- | --- | --- | --- | --- | --- | --- | --- | --- | --- | --- | --- | --- |
|  |  | **OS** | | | | **PFS** | | | | **OS** | | | | **PFS** | | | |
| **Method** | **Tumor Only** | ***C*-index** | **lower 95% CI** | **upper 95% CI** | ***P*-value** | ***C*-index** | **lower 95% CI** | **upper 95% CI** | ***P*-value** | ***C*-index** | **lower 95% CI** | **upper 95% CI** | ***P*-value** | ***C*-index** | **lower 95% CI** | **upper 95% CI** | ***P*-value** |
| ABMIL | 🗶 | 0.556 | 0.506 | 0.608 | 0.006 | 0.553 | 0.512 | 0.596 | 0.001 | 0.588 | 0.549 | 0.631 | <0.001 | 0.584 | 0.549 | 0.619 | <0.001 |
| ABMIL | ✓ | 0.593 | 0.549 | 0.640 | <0.001 | 0.587 | 0.551 | 0.623 | <0.001 | **0.597** | 0.554 | 0.644 | <0.001 | **0.603** | 0.567 | 0.641 | <0.001 |
| CLAM | 🗶 | 0.607 | 0.557 | 0.655 | <0.001 | 0.603 | 0.561 | 0.643 | <0.001 | 0.586 | 0.542 | 0.632 | <0.001 | 0.590 | 0.556 | 0.628 | <0.001 |
| CLAM | ✓ | 0.616 | 0.567 | 0.664 | <0.001 | 0.611 | 0.572 | 0.649 | <0.001 | 0.567 | 0.522 | 0.614 | 0.003 | 0.578 | 0.539 | 0.616 | <0.001 |
| AttriMIL | 🗶 | 0.609 | 0.560 | 0.660 | <0.001 | 0.597 | 0.555 | 0.639 | <0.001 | 0.580 | 0.533 | 0.627 | <0.001 | 0.586 | 0.551 | 0.622 | <0.001 |
| AttriMIL | ✓ | **0.628** | 0.584 | 0.675 | <0.001 | **0.619** | 0.583 | 0.659 | <0.001 | 0.556 | 0.511 | 0.603 | 0.011 | 0.566 | 0.530 | 0.605 | <0.001 |
| DTFD-MIL | 🗶 | 0.616 | 0.566 | 0.663 | <0.001 | 0.607 | 0.566 | 0.647 | <0.001 | 0.574 | 0.528 | 0.620 | <0.001 | 0.580 | 0.544 | 0.619 | <0.001 |
| DTFD-MIL | ✓ | 0.613 | 0.564 | 0.661 | <0.001 | 0.609 | 0.571 | 0.647 | <0.001 | 0.575 | 0.531 | 0.623 | <0.001 | 0.585 | 0.548 | 0.625 | <0.001 |
| Snuffy | 🗶 | 0.616 | 0.566 | 0.663 | <0.001 | 0.587 | 0.548 | 0.628 | <0.001 | 0.561 | 0.516 | 0.608 | 0.004 | 0.563 | 0.527 | 0.601 | 0.002 |
| Snuffy | ✓ | 0.613 | 0.564 | 0.661 | <0.001 | 0.583 | 0.545 | 0.624 | <0.001 | 0.558 | 0.512 | 0.604 | 0.007 | 0.561 | 0.524 | 0.599 | <0.001 |
| TransMIL | 🗶 | 0.613 | 0.562 | 0.663 | <0.001 | 0.606 | 0.563 | 0.646 | <0.001 | 0.580 | 0.535 | 0.627 | <0.001 | 0.587 | 0.552 | 0.624 | <0.001 |
| TransMIL | ✓ | 0.614 | 0.565 | 0.663 | <0.001 | 0.611 | 0.573 | 0.650 | <0.001 | 0.562 | 0.517 | 0.608 | 0.005 | 0.572 | 0.535 | 0.610 | <0.001 |

**Supplementary Table 8:** **Comparison of population statistics between test cohorts.** Summary statistics of patients without FISH (Clinical Test Cohort) and patients with FISH (External Test Cohort) were compared. A *P*-value greater than 0.05 indicates no significant difference between the two groups. The mean and standard deviation (SD) are reported for age and the International Prognostic Index (IPI). For the categorical variables of gender, IPI lactate dehydrogenase (LDH) indicator, IPI Eastern Cooperative Oncology Group (ECOG) performance score, and IPI stage, the corresponding number of patients and percentage are reported.

|  | |  | **Overall** | **Patients with FISH (External Test Cohort)** | **Patients without FISH (Clinical Test Cohort)** | ***P*-value** |
| --- | --- | --- | --- | --- | --- | --- |
| **n** |  | | 983 | 499 | 484 |  |
| **AGE, mean (SD)** |  | | 56.4 (15.2) | 59.3 (14.1) | 53.3 (15.7) | <0.001 |
| **GENDER, n (%)** | **0.0** | | 534 (54.3) | 270 (54.1) | 264 (54.5) | 0.941 |
|  | **1.0** | | 449 (45.7) | 229 (45.9) | 220 (45.5) |  |
| **IPI_LDH, n (%)** | **0.0** | | 542 (55.1) | 284 (56.9) | 258 (53.3) | 0.283 |
|  | **1.0** | | 441 (44.9) | 215 (43.1) | 226 (46.7) |  |
| **IPI_ECOG, n (%)** | **0.0** | | 920 (93.6) | 467 (93.6) | 453 (93.6) | 1 |
|  | **1.0** | | 63 (6.4) | 32 (6.4) | 31 (6.4) |  |
| **IPI_STAGE, n (%)** | **0.0** | | 501 (51.0) | 267 (53.5) | 234 (48.3) | 0.120 |
|  | **1.0** | | 482 (49.0) | 232 (46.5) | 250 (51.7) |  |
| **IPI, mean (SD)** |  | | 1.7 (1.2) | 1.7 (1.2) | 1.7 (1.2) | 0.610 |

**Supplementary Table 9: Correlation between MYC-R predictions and NanoString gene expression values.** Additional NanoString genes were included, along with their corresponding Pearson correlation coefficients with confidence intervals, *P*-values and adjusted *P*-values using FDR correction, to measure the correlation between the predicted score and the gene expression value. Only genes with significant correlation (62/90) are reported and sorted in descending order.

| **Gene** | **Correlation** | ***P*-value** | **95% lower CI** | **95% upper CI** | ***P*-value adjusted** |
| --- | --- | --- | --- | --- | --- |
| PTGIR | -0.379 | <0.001 | -0.451 | -0.302 | <0.001 |
| IL13RA1 | -0.362 | <0.001 | -0.435 | -0.284 | <0.001 |
| GPR137B | -0.339 | <0.001 | -0.414 | -0.260 | <0.001 |
| PDCD1LG2 | -0.338 | <0.001 | -0.412 | -0.258 | <0.001 |
| TRAF1 | -0.322 | <0.001 | -0.398 | -0.242 | <0.001 |
| IFIH1 | -0.321 | <0.001 | -0.397 | -0.240 | <0.001 |
| RHOF | -0.320 | <0.001 | -0.396 | -0.240 | <0.001 |
| NECAP2 | -0.297 | <0.001 | -0.375 | -0.216 | <0.001 |
| MYC | 0.293 | <0.001 | 0.212 | 0.371 | <0.001 |
| SNHG19 | 0.292 | <0.001 | 0.211 | 0.370 | <0.001 |
| FSCN1 | -0.282 | <0.001 | -0.360 | -0.200 | <0.001 |
| BATF3 | -0.281 | <0.001 | -0.359 | -0.199 | <0.001 |
| MST1R | -0.276 | <0.001 | -0.354 | -0.194 | <0.001 |
| BTG2 | 0.275 | <0.001 | 0.193 | 0.354 | <0.001 |
| CD80 | -0.275 | <0.001 | -0.353 | -0.192 | <0.001 |
| CARD11 | 0.261 | <0.001 | 0.179 | 0.341 | <0.001 |
| LIMA1 | -0.249 | <0.001 | -0.329 | -0.166 | <0.001 |
| SNX11 | -0.243 | <0.001 | -0.323 | -0.160 | <0.001 |
| SLAMF1 | -0.243 | <0.001 | -0.323 | -0.160 | <0.001 |
| NFIL3 | -0.239 | <0.001 | -0.319 | -0.155 | <0.001 |
| FAM159A | 0.232 | <0.001 | 0.148 | 0.313 | <0.001 |
| RFFL | -0.229 | <0.001 | -0.310 | -0.145 | <0.001 |
| SEMA7A | -0.228 | <0.001 | -0.308 | -0.143 | <0.001 |
| VASP | -0.226 | <0.001 | -0.307 | -0.142 | <0.001 |
| TMOD1 | -0.224 | <0.001 | -0.305 | -0.139 | <0.001 |
| STAT3 | -0.223 | <0.001 | -0.304 | -0.138 | <0.001 |
| CCL17 | -0.220 | <0.001 | -0.301 | -0.136 | <0.001 |
| TFPI2 | -0.220 | <0.001 | -0.301 | -0.136 | <0.001 |
| IL21R | -0.212 | <0.001 | -0.294 | -0.128 | <0.001 |
| EBI3 | -0.210 | <0.001 | -0.292 | -0.125 | <0.001 |
| S1PR2 | -0.195 | <0.001 | -0.277 | -0.110 | <0.001 |
| QSOX1 | -0.189 | <0.001 | -0.271 | -0.103 | <0.001 |
| QRSL1 | 0.186 | <0.001 | 0.100 | 0.268 | <0.001 |
| UBXN4 | -0.182 | <0.001 | -0.265 | -0.097 | <0.001 |
| BANK1 | 0.172 | <0.001 | 0.086 | 0.255 | <0.001 |
| TNFSF8 | 0.171 | <0.001 | 0.085 | 0.254 | <0.001 |
| TNFRSF13B | 0.165 | <0.001 | 0.079 | 0.249 | <0.001 |
| LY75 | -0.163 | <0.001 | -0.246 | -0.077 | <0.001 |
| BCL2 | 0.162 | <0.001 | 0.076 | 0.245 | <0.001 |
| CD24 | 0.159 | <0.001 | 0.073 | 0.243 | <0.001 |
| MAML3 | -0.158 | <0.001 | -0.241 | -0.072 | <0.001 |
| LIMD1 | 0.151 | <0.001 | 0.065 | 0.235 | 0.001 |
| MOBKL2C | -0.151 | <0.001 | -0.235 | -0.065 | 0.001 |
| OR13A1 | 0.148 | <0.001 | 0.062 | 0.232 | 0.002 |
| PEG10 | 0.144 | 0.001 | 0.057 | 0.228 | 0.002 |
| PRKCB | 0.142 | 0.001 | 0.056 | 0.226 | 0.003 |
| GSK3B | -0.138 | 0.002 | -0.223 | -0.052 | 0.003 |
| PRDX2 | 0.133 | 0.003 | 0.047 | 0.218 | 0.005 |
| RCL1 | -0.133 | 0.003 | -0.217 | -0.046 | 0.005 |
| R3HDM1 | 0.131 | 0.003 | 0.045 | 0.216 | 0.005 |
| OPA1 | 0.127 | 0.004 | 0.040 | 0.211 | 0.007 |
| ASB13 | -0.125 | 0.005 | -0.209 | -0.038 | 0.008 |
| SGPP2 | -0.120 | 0.007 | -0.204 | -0.033 | 0.012 |
| PIM2 | 0.116 | 0.009 | 0.029 | 0.201 | 0.015 |
| ITPKB | -0.115 | 0.010 | -0.200 | -0.028 | 0.016 |
| CCDC50 | 0.105 | 0.018 | 0.018 | 0.190 | 0.029 |
| SERPINA9 | -0.101 | 0.022 | -0.187 | -0.015 | 0.035 |
| MIR155HG | -0.099 | 0.026 | -0.184 | -0.012 | 0.040 |
| SMIM14 | 0.095 | 0.033 | 0.008 | 0.180 | 0.050 |
| JCHAIN | 0.090 | 0.043 | 0.003 | 0.175 | 0.064 |
| WDR55 | -0.088 | 0.046 | -0.174 | -0.002 | 0.068 |
| AUH | -0.088 | 0.047 | -0.174 | -0.001 | 0.068 |

**Supplementary Table 10: Correlation between MYC-R predictions and Gene expression values.** Genes from the lymphoma-associated macrophage interaction signature (LAMIS)^12^ were used to measure the correlation between predicted score and gene expression value. The resulting Pearson correlation coefficient with confidence intervals, *P*-values and adjusted *P*-values using FDR correction are provided. Only genes with significant correlation (76/145) are reported and sorted in descending order.

| **Gene** | **Correlation** | ***P*-value** | **95% lower CI** | **95% upper CI** | ***P*-value adjusted** |
| --- | --- | --- | --- | --- | --- |
| ITGB2 | -0.338 | <0.001 | -0.440 | -0.227 | <0.001 |
| LAMB2 | -0.297 | <0.001 | -0.402 | -0.184 | <0.001 |
| LGALS1 | -0.293 | <0.001 | -0.399 | -0.180 | <0.001 |
| HK3 | -0.292 | <0.001 | -0.398 | -0.178 | <0.001 |
| C1S | -0.289 | <0.001 | -0.395 | -0.175 | <0.001 |
| POLH | 0.285 | <0.001 | 0.171 | 0.391 | <0.001 |
| TLR9 | 0.285 | <0.001 | 0.171 | 0.391 | <0.001 |
| DPYSL3 | -0.285 | <0.001 | -0.391 | -0.171 | <0.001 |
| CSF1R | -0.282 | <0.001 | -0.388 | -0.168 | <0.001 |
| TYMP | -0.278 | <0.001 | -0.385 | -0.164 | <0.001 |
| FN1 | -0.274 | <0.001 | -0.381 | -0.159 | <0.001 |
| FBN1 | -0.271 | <0.001 | -0.378 | -0.156 | <0.001 |
| ACOT1 | -0.267 | <0.001 | -0.375 | -0.153 | <0.001 |
| ADAMDEC1 | -0.258 | <0.001 | -0.366 | -0.143 | <0.001 |
| SERPING1 | -0.257 | <0.001 | -0.365 | -0.142 | <0.001 |
| LYZ | -0.255 | <0.001 | -0.364 | -0.140 | <0.001 |
| MFAP2 | -0.254 | <0.001 | -0.363 | -0.139 | <0.001 |
| TGFBI | -0.252 | <0.001 | -0.361 | -0.137 | <0.001 |
| CD14 | -0.250 | <0.001 | -0.359 | -0.135 | <0.001 |
| c13orf18 | 0.248 | <0.001 | 0.132 | 0.357 | <0.001 |
| HSPG2 | -0.247 | <0.001 | -0.356 | -0.132 | <0.001 |
| ITGAM | -0.247 | <0.001 | -0.356 | -0.131 | <0.001 |
| CD80 | -0.246 | <0.001 | -0.355 | -0.130 | <0.001 |
| CSF1 | -0.246 | <0.001 | -0.356 | -0.131 | <0.001 |
| CD68 | -0.244 | <0.001 | -0.353 | -0.128 | <0.001 |
| DCN | -0.241 | <0.001 | -0.350 | -0.125 | <0.001 |
| GSN | -0.239 | <0.001 | -0.349 | -0.123 | <0.001 |
| CAPG | -0.236 | <0.001 | -0.346 | -0.120 | <0.001 |
| SERPINA1 | -0.232 | <0.001 | -0.342 | -0.116 | <0.001 |
| TIMP1 | -0.229 | <0.001 | -0.339 | -0.112 | <0.001 |
| COL1A1 | -0.228 | <0.001 | -0.339 | -0.112 | <0.001 |
| CD81 | -0.228 | <0.001 | -0.339 | -0.112 | <0.001 |
| NASP | 0.219 | <0.001 | 0.102 | 0.330 | 0.001 |
| ITGAX | -0.218 | <0.001 | -0.329 | -0.101 | 0.001 |
| LIG3 | 0.214 | <0.001 | 0.096 | 0.325 | 0.002 |
| CTSB | -0.214 | <0.001 | -0.325 | -0.097 | 0.002 |
| MDM4 | 0.209 | <0.001 | 0.092 | 0.321 | 0.002 |
| CTSH | -0.207 | <0.001 | -0.319 | -0.090 | 0.002 |
| CD274 | -0.205 | <0.001 | -0.317 | -0.087 | 0.003 |
| TCF3 | 0.204 | <0.001 | 0.087 | 0.316 | 0.003 |
| IL34 | -0.203 | <0.001 | -0.315 | -0.086 | 0.003 |
| BCL2A1 | -0.193 | 0.001 | -0.306 | -0.075 | 0.005 |
| CLU | -0.192 | 0.002 | -0.305 | -0.074 | 0.005 |
| LAP3 | -0.192 | 0.002 | -0.304 | -0.074 | 0.005 |
| TEK | -0.189 | 0.002 | -0.302 | -0.071 | 0.006 |
| CXCR4 | 0.187 | 0.002 | 0.069 | 0.300 | 0.007 |
| BRI3BP | 0.187 | 0.002 | 0.069 | 0.300 | 0.006 |
| IL18 | -0.186 | 0.002 | -0.299 | -0.068 | 0.007 |
| MMP9 | -0.185 | 0.002 | -0.298 | -0.067 | 0.007 |
| TMSB4X | -0.184 | 0.002 | -0.297 | -0.066 | 0.007 |
| SH3BP5 | 0.184 | 0.002 | 0.066 | 0.297 | 0.007 |
| RNASEH2B | 0.177 | 0.004 | 0.059 | 0.291 | 0.01 |
| CD40 | -0.175 | 0.004 | -0.288 | -0.056 | 0.011 |
| CR2 | -0.174 | 0.004 | -0.288 | -0.056 | 0.011 |
| SIT1 | 0.174 | 0.004 | 0.056 | 0.288 | 0.011 |
| CD84 | -0.173 | 0.005 | -0.286 | -0.054 | 0.012 |
| SIGLEC10 | -0.170 | 0.005 | -0.284 | -0.052 | 0.013 |
| SERPINB1 | -0.170 | 0.005 | -0.284 | -0.052 | 0.013 |
| BAFF | -0.169 | 0.005 | -0.283 | -0.050 | 0.013 |
| CTCF | 0.168 | 0.006 | 0.049 | 0.282 | 0.014 |
| CD44 | -0.164 | 0.007 | -0.278 | -0.045 | 0.017 |
| PHPT1 | -0.164 | 0.007 | -0.278 | -0.045 | 0.017 |
| APOBEC3A | -0.163 | 0.007 | -0.278 | -0.045 | 0.017 |
| EIF2B4 | 0.161 | 0.008 | 0.043 | 0.276 | 0.018 |
| TNFRSF4 | -0.158 | 0.009 | -0.273 | -0.040 | 0.021 |
| SLC7A5 | 0.158 | 0.009 | 0.039 | 0.272 | 0.021 |
| TPD52 | 0.155 | 0.011 | 0.036 | 0.269 | 0.024 |
| AICDA | 0.155 | 0.011 | 0.036 | 0.269 | 0.024 |
| RORC1 | -0.154 | 0.011 | -0.269 | -0.035 | 0.024 |
| POLR2B | 0.153 | 0.012 | 0.034 | 0.268 | 0.024 |
| CD163 | -0.146 | 0.016 | -0.261 | -0.027 | 0.033 |
| PTGDS | -0.146 | 0.017 | -0.261 | -0.027 | 0.034 |
| ATM | 0.143 | 0.019 | 0.024 | 0.258 | 0.038 |
| CCL18 | -0.140 | 0.022 | -0.255 | -0.020 | 0.043 |
| IGLJ3 | 0.139 | 0.023 | 0.019 | 0.254 | 0.044 |
| CD47 | 0.137 | 0.025 | 0.018 | 0.252 | 0.047 |

**Supplementary References**

1.	Huttner, M. Pamly. (2025).

2.	Chen, R. J. *et al.* Towards a general-purpose foundation model for computational pathology. *Nat. Med.* **30**, 850–862 (2024).

3.	Ilse, M., Tomczak, J. & Welling, M. Attention-based deep multiple instance learning. in *International conference on machine learning* 2127–2136 (2018).

4.	Raffel, C. & Ellis, D. P. W. Feed-forward networks with attention can solve some long-term memory problems. *ArXiv Prepr. ArXiv151208756* (2015).

5.	Lin, Z. *et al.* A structured self-attentive sentence embedding. *ArXiv Prepr. ArXiv170303130* (2017).

6.	Lu, M. Y. *et al.* Data-efficient and weakly supervised computational pathology on whole-slide images. *Nat. Biomed. Eng.* **5**, 555–570 (2021).

7.	Zhang, H. *et al.* Dtfd-mil: Double-tier feature distillation multiple instance learning for histopathology whole slide image classification. in *Proceedings of the IEEE/CVF conference on computer vision and pattern recognition* 18802–18812 (2022).

8.	Cai, L., Huang, S., Zhang, Y., Lu, J. & Zhang, Y. Rethinking Attention-Based Multiple Instance Learning for Whole-Slide Pathological Image Classification: An Instance Attribute Viewpoint. *ArXiv Prepr. ArXiv240400351* (2024).

9.	Qian, Z. *et al.* Transformer based multiple instance learning for weakly supervised histopathology image segmentation. in *International Conference on Medical Image Computing and Computer-Assisted Intervention* 160–170 (2022).

10.	Jafarinia, H., Alipanah, A., Razavi, S., Mirzaie, N. & Rohban, M. H. Snuffy: Efficient Whole Slide Image Classifier. in *European Conference on Computer Vision* 243–260 (2024).

11.	Loshchilov, I. & Hutter, F. Decoupled weight decay regularization. *ArXiv Prepr. ArXiv171105101* (2017).

12.	Staiger, A. M. *et al.* A novel lymphoma-associated macrophage interaction signature (LAMIS) provides robust risk prognostication in diffuse large B-cell lymphoma clinical trial cohorts of the DSHNHL. *Leukemia* **34**, 543–552 (2020).
